# Supplementary material for: Validation of the German Life-Space Assessment (LSA-D): cross-sectional validation study in urban and rural community-dwelling older adults
Source: BMJ Open. 2021 Jul 6;11(7):e049926. doi: 10.1136/bmjopen-2021-049926 (PMC8261868; doi:10.1136/bmjopen-2021-049926)
Supplement: Supplementary data [file bmjopen-2021-049926supp001.pdf]

German Life-Space-Assessment LSA-D

**DIE NÄCHSTEN FRAGEN BEZIEHEN SICH AUF IHRE AKTIVITÄTEN IN DEN LETZTEN VIER WOCHEN:**			**A. WIE HÄUFIG WAREN SIE IN DEN LETZTEN 4 WOCHEN IN (Name des Life-Space-Levels)? Häufigkeit**				**WIE SIND SIE DORT HINGEKOMMEN? B. HABEN SIE HILFSMITTEL ODER ANDERE AUSRÜSTUNG DAFÜR VERWENDET?**			**C. BENÖTIGTEN SIE DAFÜR DIE HILFE EINER ANDEREN PERSON?**		
WAREN SIE IN DEN LETZTEN 4 WOCHEN…	Ja	Nein	Weniger als 1 mal die Woche	1-3 mal die Woche	4-6 mal die Woche	Täglich	Ja	Nein	Unbekannt / keine Angabe	Ja	Nein	Unbekannt / keine Angabe
IN ANDEREN RÄUMEN IHRES ZUHAUSES außer dem Raum, in dem Sie schlafen? *LIFE-SPACE 1*	**LS1** ❑	❑	**LS1-Häufigkeit** ❑	❑	❑	❑	**LS1-Hilfsmittel** ❑	❑	❑	**LS1-Persönliche Hilfe** ❑	❑	❑
IN DER NÄHEREN UMGEBUNG AUSSERHALB IHRER WOHNUNG (Hausflur, Terrasse, Balkon, Fahrstuhl, Hof, Garage, hauseigener Garten, Auffahrt)? *LIFE-SPACE 2*	**LS2** ❑	❑	**LS2-Häufigkeit** ❑	❑	❑	❑	**LS2-Hilfsmittel** ❑	❑	❑	**LS2-Persönliche Hilfe** ❑	❑	❑
AN ORTEN IN IHRER NACHBARSCHAFT, aber außerhalb Ihrer Wohnung oder Ihrer näheren Wohnumgebung? *LIFE-SPACE 3*	**LS3** ❑	❑	**LS3-Häufigkeit** ❑	❑	❑	❑	**LS3-Hilfsmittel** ❑	❑	❑	**LS3-Persönliche Hilfe** ❑	❑	❑
AN ORTEN AUSSERHALB IHRER NACHBARSCHAFT, aber innerhalb der Stadt oder der Ortschaft, in der Sie leben? *LIFE-SPACE 4*	**LS4** ❑	❑	**LS4-Häufigkeit** ❑	❑	❑	❑	**LS4-Hilfsmittel** ❑	❑	❑	**LS4-Persönliche Hilfe** ❑	❑	❑
AN ORTEN AUSSERHALB DER STADT ODER DER ORTSCHAFT, in der Sie leben? *LIFE-SPACE 5*	**LS5** ❑	❑	**LS5-Häufigkeit** ❑	❑	❑	❑	**LS5-Hilfsmittel** ❑	❑	❑	**LS5-Persönliche Hilfe** ❑	❑	❑
