# Supplementary material for: Validation of the German Life-Space Assessment (LSA-D): cross-sectional validation study in urban and rural community-dwelling older adults
Source: BMJ Open. 2021 Jul 6;11(7):e049926. doi: 10.1136/bmjopen-2021-049926 (PMC8261868; doi:10.1136/bmjopen-2021-049926)
Supplement: Author's manuscript [file bmjopen-2021-049926.draft_revisions.pdf]

BMJ Open

BMJ Open is committed to open peer review. As part of this commitment we make the peer review history of every article we publish publicly available.

When an article is published we post the peer reviewers' comments and the authors' responses online. We also post the versions of the paper that were used during peer review. These are the versions that the peer review comments apply to.

The versions of the paper that follow are the versions that were submitted during the peer review process. They are not the versions of record or the final published versions. They should not be cited or distributed as the published version of this manuscript.

BMJ Open is an open access journal and the full, final, typeset and author-corrected version of record of the manuscript is available on our site with no access controls, subscription charges or pay-per-view fees (http://bmjopen.bmj.com).

If you have any questions on BMJ Open's open peer review process please email info.bmjopen@bmj.com

BMJ Open

Validation of the German version of the Life-Space Assessment LSA-D

Journal:	*BMJ Open*
Manuscript ID	bmjopen-2021-049926
Article Type:	Original research
Date Submitted by the Author:	10-Feb-2021
Complete List of Authors:	Mümken, Sandra Angelika; Charité Universitätsmedizin Berlin, 1 Institute of Medical Sociology and Rehabilitation Science<br>Gellert, Paul; Charité Universitätsmedizin Berlin, Institute of Medical Sociology and Rehabilitation Science<br>Stollwerck, Malte; Charite Universitatsmedizin Berlin, Institute of Medical Sociology and Rehabilitation Science<br>O'Sullivan, Julie Lorraine; Charite Universitatsmedizin Berlin, Institute of Medical Sociology and Rehabilitation Science<br>Kiselev, Joern; Charite Universitatsmedizin Berlin, Department of Anesthesiology and Operative Intensive Care Medicine (CCM, CVK)
Keywords:	Rehabilitation medicine < INTERNAL MEDICINE, GERIATRIC MEDICINE, SOCIAL MEDICINE

SCHOLARONE™
Manuscripts

I, the Submitting Author has the right to grant and does grant on behalf of all authors of the Work (as defined in the below author licence), an exclusive licence and/or a non-exclusive licence for contributions from authors who are: i) UK Crown employees; ii) where BMJ has agreed a CC-BY licence shall apply, and/or iii) in accordance with the terms applicable for US Federal Government officers or employees acting as part of their official duties; on a worldwide, perpetual, irrevocable, royalty-free basis to BMJ Publishing Group Ltd ("BMJ") its licensees and where the relevant Journal is co-owned by BMJ to the co-owners of the Journal, to publish the Work in this journal and any other BMJ products and to exploit all rights, as set out in our licence.

The Submitting Author accepts and understands that any supply made under these terms is made by BMJ to the Submitting Author unless you are acting as an employee on behalf of your employer or a postgraduate student of an affiliated institution which is paying any applicable article publishing charge ("APC") for Open Access articles. Where the Submitting Author wishes to make the Work available on an Open Access basis (and intends to pay the relevant APC), the terms of reuse of such Open Access shall be governed by a Creative Commons licence – details of these licences and which Creative Commons licence will apply to this Work are set out in our licence referred to above.

Other than as permitted in any relevant BMJ Author's Self Archiving Policies, I confirm this Work has not been accepted for publication elsewhere, is not being considered for publication elsewhere and does not duplicate material already published. I confirm all authors consent to publication of this Work and authorise the granting of this licence.

For peer review only

Validation of the German version of the Life-Space Assessment LSA-D

Sandra A. Mümken[1]

Paul Gellert[1]

Malte Stollwerck[1]

Julie L. O'Sullivan[1]

Jörn Kiselev[2]

[1] *Institute of Medical Sociology and Rehabilitation Science, Charité – Universitätsmedizin Berlin, Germany, corporate member of Freie Universität Berlin, Humboldt-Universität zu Berlin, and Berlin Institute of Health, Charitéplatz 1, D-10117 Berlin, Germany*

[2] *Department of Anesthesiology and Operative Intensive Care Medicine (CCM, CVK), Charité Universitätsmedizin Berlin, corporate member of Freie Universität Berlin, Humboldt-Universität zu Berlin, and Berlin Institute of Health, Charitéplatz 1, D-10117 Berlin, Germany*

Corresponding author:

Sandra A. Mümken

Institute of Medical Sociology and Rehabilitation Science

Charitéplatz 1 10117 Berlin

sandra.muemken@charite.de

Wordcount:3.995

For peer review only

Abstract

Objectives To develop a German version of the original University of Alabama at Birmingham (UAB) Study of Aging Life-Space Assessment (LSA-D) for measurement of community mobility in older adults within the past 4 weeks and to evaluate its psychometric properties for urban and rural populations of older adults.

Design Cross-sectional validation study.

Setting Two study centres in urban and rural German outpatient hospital settings.

Participants In total N=83 community-dwelling older adults were recruited (n=40 from urban and n=43 from rural areas; mean age was 78.5 (SD=5.4); 49% male).

Primary and secondary outcome measures The final version of the translated LSA-D was related with questions about activities and instrumental activities of daily living (ADL/iADL; primary hypothesis), Timed-Up&Go-Test (TUG), self-rated health, balance confidence and history of falls and sociodemographic factors to obtain construct validity. Secondary outcome measures of health included handgrip strength, screening of cognitive function, comorbidities and use of transportation. To assess construct validity, correlations between LSA-D and all primary outcome measures were examined for total sample, urban and rural subsamples using bivariate regression and multiple adjusted regression models. Posthoc analyses included different LSA-D scoring methods for each region. All parameters were estimated using non-parametric bootstrapping procedure.

Results In the multiple adjusted model for the total sample, number of ADL/iADL limitations (β=-.26; 95%CI=-.42/-.08), TUG (β=-.37; 95%CI=-.68/-.14), living in shared living arrangements (β=.22; 95%CI=.01/.44) and history of falls in the past 6 months (β=-.22; 95%CI=-.41/-.05) showed significant associations with the LSA-D composite score, while living in urban area (β=-.19; 95%CI=-.42/.03) and male gender (β=.15; 95%CI=-.04/.35) were not significant.

For peer review only

Conclusion The LSA-D is a valid tool for measuring life-space mobility in German community-dwelling older adults within the past four weeks in ambulant urban and rural settings.

Trial registration number DRKS00019023

Keywords: Older adults; Mobility; Life-space; German; Validation; living environment; geriatrics,

Strengths and limitations of this study

- German validation of the original UAB Life-Space Assessment (LSA-D) for community dwelling older adults in urban and rural settings
- Using bootstrapped bivariate and multiple adjusted regression models to attain construct validity of the LSA-D
- Recruitment had to be stopped shortly before reaching the calculated sample size due to the decision to restrict in face-to-face research to contain the outbreak of the Covid-19 pandemic in March 2020

For peer review only

Introduction

Mobility, defined as "the ability to move oneself (either independently or by using assistive devices or transportation) within environments that expand from one's home to the neighbourhood and regions beyond"[1] encompasses general independence, opportunities for social activities and freedom to experience new sites. This broad concept of mobility goes beyond the narrow conception of mobility as performance in a single functional test without considering environmental barriers and social resources although their impact on mobility has been investigated.[2, 3] Therefore, the focus on single functional mobility tests can lead to misconceptions about actual mobility performance in everyday life and health practitioners may oversee possible consequences for social participation and mental health.[4]

To overcome these shortcomings of functional mobility assessments, recent studies of mobility and aging operationalize mobility as circled areas, so-called life-spaces, that spread from the centre of one's own house and garden to the neighbourhood, the city lived in and beyond, with each life-space offering different opportunities for social involvement, recreational activities or access to medical care.[5, 6] The application of self-reported life-spaces to determine mobility of older adults was first established by May et al. in 1985[7] and assessment of life-space mobility with standardized questionnaires was recently recommended for geriatric research.[8] Several instruments for measuring life-space mobility in specific populations and settings exist, including assessments of life-space within one's own residence for home-bound individuals[9] or residents in nursing homes and other institutions.[10, 11]

One of the most frequently applied instruments for measurement of mobility in older adults using the life-space concept is the validated Life-Space Assessment (LSA) by Baker et al.[12] as part of the University of Alabama at Birmingham (UAB). Study of Aging. The LSA provides health professionals in geriatric settings with information on availability of environmental and

For peer review only

social resources as an outcome of mobility assessment and gives them a more comprehensive picture of the patient's needs.

The importance of the LSA for clinical practice has been shown in various studies. Kennedy et al.[13] for instance found that a decline in life-space over six months is associated with greater mortality in the following six months. Limitations in life-space mobility are associated with long-term mortality of older men[14], cognitive decline[15], risk of falls[16], frailty[17] and hospital admission in older adults with heart failure.[18] Furthermore, the concept has already been established in outpatient physical therapy with various community-dwelling neurological orthopaedic and surgery patients.[19] Additionally, psychological health factors like external control believes[20] and personal activity goals[21] influence life-space mobility. Therefore, the LSA can also supplement evaluation concepts in psychological research and treatment of older adults. The construct validity of the LSA was commonly tested by relating the LSA to activities an instrumental activities of daily living (ADL/iADL) but also self-rated health and fears of falling.[22, 23, 24] Moreover, as Baker et al.[12] pointed out, there is a need to validate the LSA in urban and rural settings. As part of validity testing, the LSA has been translated into multiple languages such as Chinese[25], French[24], Spanish[22], Swedish[26] or Danish[27]. To date, two modified German versions for assessment of life-space mobility in specific populations of older adults exist: One for those with mild cognitive impairment (LSA-CI)[23] that captures life-space of the past week and one for those in institutionalized settings where life-spaces are adapted to the living environment of care facilities (LSA-IS).[11] However, a validated and intercultural adapted version of the original LSA that can be administered in the context of a more general geriatric setting in the overall population of community-dwelling older adults is still missing. Therefore, we conducted a validation study of a German version of the original LSA the (LSA-D) in urban and rural areas.

Aims and Hypotheses

Our aim was to translate, apply and validate the LSA-D, a German version of the LSA from the University of Alabama (UAB) Study of Aging for the population of urban and rural community-dwelling older adults. In line with the original validation of the LSA we hypothesized a moderate association of the LSA-D composite score with limitations in ADL/iADL as primary hypothesis[12] Further, we assumed moderate associations with Timed-Up&Go-Test (TUG), self-rated health and history of falls. Finally, we expected the newly translated LSA-D to show these associations in both urban and rural populations of older adults.

Methods

Study design

A cross-sectional study design was used with two German hospital clinics as study centres. The first study centre was an ambulant geriatric rehabilitation facility of the Havelland clinics located in a small town (16,000 inhabitants) in Brandenburg, Germany. The second centre was based at the Charité – Universitätsmedizin Berlin within the Department of Anesthesiology and Operative Intensive Care. Approvement for the study was given by the local Ethics Committee of the Charité – Universitätsmedizin Berlin (EA2/124/19) and the study was prospectively registered at the German Clinical Trials Register (DRKS00019023). Sample size calculation was based on assumptions to find a moderate-to-strong association of beta/r=-.40[12] between the LSA-D-composite score and ADL/iADL (i.e., primary hypothesis), functional mobility with TUG, self-rated health and balance confidence in all observed populations. For testing of the primary hypothesis, 92 participants or 46 subjects per setting (i.e., urban/rural) were required. This was based on the following assumptions: An effect size of Pearson's correlation coefficient or standardized beta coefficient of r/beta=-0.40 (ρ=-0.40 in

For peer review only - http://bmjopen.bmj.com/site/about/guidelines.xhtml

the population) was assumed in reference to the association between the LSA composite score (LS-C) and ADL/iADL found in the original validation study of LSA.[12] The power calculation with GPower 3.1 for bivariate correlations (test family "exact")[28] resulted in an estimated minimum sample size of n=46 participants per setting (urban/rural) and a critical r=-0.29 with a type I error rate of alpha=0.025 (test one-sided; corrected for multiple testing [setting urban/rural; alpha=0.05/2]) and a statistical power of 1-β=0.80. Recruitment took place from November 2019 to February 2020 and ended in March 2020 with a sample size of 82 due to restrictions of the then starting coronavirus pandemic. A post hoc sensitivity analysis suggests that we are still able to detect effects of r=-.30 and larger.

Translation process

In accordance with the 2008 guidelines of the World Health Organization[29], forward translation into German language was separately conducted by two researchers who formulated two German versions that were discussed and then merged into one German pre-version of the LSA-D. The pre-version was given to two native English speakers for back translation. Again, both versions of the back translation were discussed by the two native speakers and a concerted version of the back-translation was produced. Differences between the original LSA and the concerted back-translation were discussed and reviewed with the original author of the LSA to redefine a pre-final version of the LSA-D that was pre-tested for understandability using cognitive interview technique among 4 older adults of the Charité – Universitätsmedizin Berlin to create the final LSA-D version.[30]

For peer review only

Participants and Recruitment

The 83 participants were divided into two groups based on the size of their place of residence. Participants from villages (< 5,000 inhabitants) and small towns (up to 40,000 inhabitants) were classified as living in rural areas. In contrast, participants who lived in the city of Berlin (3.8 million inhabitants) with its metropolitan infrastructure and services were classified as urban population. Inclusion criteria were defined as: age of 70 years and older; ability to read and understand the questionnaire and give written informed consent. Exclusion criteria were incidences that limited mobility within the past four weeks, severe cognitive limitations or mental conditions, need of acute care and insufficient understanding of the German language. In total, 126 persons were screened for eligibility of which 28 did not fulfil the inclusion criteria and 15 were unwilling to participate. In both study centres, participants were recruited during normal health care routine by trained study staff. All participants received verbal and written information on the study and were given time to consider participation before giving written consent.

Measures

Sociodemographic measures

Demographic variables (i.e., age, gender, height, weight, status of shared living-arrangements) use of public transportation and driving-status were assessed with a standardized questionnaire.

Primary outcome measures

Selection of other primary and secondary variables for determining construct validity was based on the original validation study of the UAB and other LSA validation studies from different countries.[12, 22, 23, 24]

Life-space was evaluated with the translated German Version of the UAB Life-Space Assessment. The LSA consists of a questionnaire on five different life-spaces capturing six

For peer review only

possible levels of life-space (0. Mobility within the bedroom, 1. rooms inside the home besides the bedroom, 2. area outside the house, 3. neighbourhood, 4. town or city lived in, outside of town or city lived in). For each level, participants were asked a) if they went to this level in the past four weeks, b) if so, how often, c) if they needed assistive devices or special equipment to reach that level and d) if they needed personal help to reach that level.[12] Different scoring methods can be used with the LSA either indicating the maximum attained life-space level (LS-M), life-space that can be reached independently without any further support (LS-I), reachable life-space with possible use of equipment but without personal help (LS-E), dichotomized life-space (LS-D) that classifies a person's mobility into the ability to travel beyond the borders of their self-perceived neighbourhood and the composite score (LS-C) that summarizes the attained LS-level, needed equipment or personal support and frequency of visits. The LS-C score ranges from 0 to 120 points with higher scores indicating better mobility. As the LS-C score has shown a good sensitivity regarding change over time, it is frequently applied in longitudinal studies.[31, 32] In cross-sectional studies, LS-I and LS-D are additional scores for describing actual mobility and associations with other health factors.[12]

Limitations in ADL and iADL were investigated using questions from the "Survey of Aging and Retirement in Europe" (SHARE).[33] Participants were asked whether they had difficulties due to physical, emotional or cognitive problems to perform 15 activities like dressing, gardening, using a map or making a telephone call. Binary response options for each activity were yes or no. Subsequently, a sum score of limitations in ADL/iADL activities was calculated ranging from 0 to 15. Higher scores indicate more functional impairments.

The TUG is one of the most frequently used measures of balance and functional mobility in older adults and is a recommended tool for geriatric assessment.[34] During performance of the TUG, time (in seconds) is taken for rising up from a standardized chair, walking three metres, turning around, walking back and siting down again in a comfortable self-selected

For peer review only

speed.[35] Higher TUG times are associated with stronger mobility and ADL restrictions.[36, 37]

The EQ-VAS from the EQ-5D-5L version was used to record overall self-rated health of the day on a vertical visual analogue scale ranging from 0 points for the worst imaginable health to 100 points for the best conceivable health.[38] To measure balance confidence, we used the ABC-6-Scale that was translated into German and validated by Schott et al.[39] Participants were accounted to have a history of falls if they had fallen at least one time in the past six months using the criteria's of the "Frailty and Injuries: Cooperative Studies of Intervention Techniques" to define a fall[40].

Secondary outcome measures of health

Hand grip strength was measured as maximum of three contractions with a hydraulic handheld dynamometer (Sahean SH5001; Changwon, South Korea) in the dominant hand and standardized sitting position.[41] We administered the Charlson comorbidity index (CCI) as a method to categorize comorbidities (0-41 points) where scores of >5 indicate a higher mortality risk.[42] Cognitive status was assessed with the Mini-Cog screening tool where a score ranging from 0-5 can be achieved and a score of 0-2 is seen as an indicator for further investigation of cognitive status.[43]

Statistical analysis

Means (M) and standard deviations (SD) were reported descriptively for continuous demographic variables (i.e., age, height, weight) and health measures (i.e., limitations in ADL/iADL, time in seconds needed to complete TUG, self-rated health and balance confidence). Gender, status of shared living arrangements, use of different transportation modes and history of falls were reported for the total and each subsample as absolute frequencies and percentage of participants. Distribution of the data was skewed therefore we used the non-parametric, bias corrected and accelerated (BCA) bootstrap method with 10,000 resamples and

For peer review only

fixed random seeds that resamples the collected data with replacement to derive robust results.[44] With the BCA bootstrap method, coefficients and confidence intervals can be estimated with good statistical power even if sample sizes are small and distribution of data is unknown or not normal. For investigating differences between urban and rural participants, the Welch Test was performed as it has been recommended as a standard test for small samples.[45] To determine construct validity of the LSA-D, BCA bootstrap method and standardized z-scores (i.e., that can be interpreted like beta coefficients) of the included binary and continuous variables (i.e., age, male gender, rural or urban residence, status of shared living arrangements, sum score of limitations with ADL/iADL activities, functional mobility with TUG, self-rated health, balance confidence and history of falls) were used for bivariate and multivariate regression analysis. Scores of the ABC-6 scale had to be excluded from multivariate regression because they revealed a correlation of r=-.72 with TUG scores. To avoid multicollinearity, it was decided to include only the TUG score due to its importance as a physical measurement of functional mobility for assessing construct validity. All analyses were run using SPSS version 25. Microsoft Excel 2016 was used to create the figure.

Results

Sample characteristics

For the total sample (N=83), mean age was 78.5 (SD=5.4) years and about half of the sample (n=41; 49.4%) were male. 47 participants (56.6%) lived together with others in a shared living arrangement. In the past four weeks, 39 participants (47.0%) drove a car by themselves, 18 participants (21.7%) rode a bicycle and 34 participants (41.0%) used walking aids. On average, participants had a TUG of M=13.90 (SD=9.20) seconds. Score of limitations in ADL/iADL was moderate with M=7.8 (SD=6.2) and mean score of self-rated health was M=64.7 (SD=21.3).

For peer review only

When comparing urban with rural participants, those living in urban areas had significantly more ADL/iADL limitations, t(74.51)=-2.34; p=.022, and comorbidities, t(57.27)=-2.44; p=.018. Rural participants were significantly older, t(81)=2.43; p=.017, needed more time to complete the TUG, t(70.65)=3.33; p=.001, had less balance confidence, t(80.11)=-2.84; p=.006, were more often assigned to a care level, t(69.62)=4.53; p<.001, and had lower self-rated health, t(81)=-2.45; p=.016. Concerning the utilization of means of transportation, the percentage of participants who drove a car or a bicycle for independent mobility within the last 4 weeks did not differ significantly across regions. Characteristics of participants in total and separately for each region are presented in table 1.

For peer review only

Table 1: Participant Characteristics

Variable	total (N=83)		urban (*n*=40)		rural (*n*=43)	
	N	*%*	*N*	*%*	*N*	*%*
Gender (male)	41	49.4	23	57.5	18	41.9
Status of shared living arrangements	47	56.6	19	47.5	28	65.1
Drove a car in past 4 weeks	39	47.0	18	45.0	21	48.8
Rode a bicycle in past 4 weeks	18	21.7	9	22.5	9	20.9
Used walking aid in past 4 weeks	34	41.0	11	27.5	23	53.5
History of falls past 6 months (>1)	22	26.5	9	22.5	13	30.2
	M	*SD*	*M*	*SD*	*M*	*SD*
Age (years)	78.5	5.4	77.1	5.2	79.8	5.2
Height (cm)	168.7 (3)	10.5	170.8	11.2	166.5 (3)	9.3
Weight (kg)	79.0 (3)	18.8	79.7	19.8	78.3 (3)	17.8
Body Mass Index	27.6 (3)	5.4	27.1	5.8	28.1 (3)	5.1
Charlson Comorbidity Index (0-41)	3.2	3.6	4.2	4.5	2.3	2.3
Hand grip strength (kg)	25.8 (2)	11.6	27.7 (1)	11.8	24.0 (1)	11.4
Mini-Cog™-Score (0-5)	3.8	1.5	3.8	1.6	3.8	1.4
LSA-D composite score (0-120)	60.8	24.3	58.0	21.7	63.3	26.5
ADL/iADL (number of limitations; 0-15)	7.8	6.2	9.4	6.7	6.2	5.4
TUG (s)	13.9 (5)	9.2	10.5 (3)	6.8	16.9 (2)	10.1
Self-rated health (0-100)	64.8	21.3	70.5	19.3	59.4	21.8
Balance confidence (0-100)	57.8	32.7	67.8	28.4	48.4	34.0

Note: Live-Space-Assessment-Deutsch (LSA-D); Activities of Daily Living (ADL); Instrumented Activities of Daily Living (iADL);
Timed-Up &Go-Test (TUG); Numbers in brackets report number of missing values

For peer review only

Descriptive statistics of the LSA-D

Life-space-level 5, as maximum life-space (LS-M), was reached by 60.2% of the total sample. 32.5% of all participants had an independent life-space level (LS-I) of 5 while the remaining needed either equipment or personal help. For the urban subsample, 40.0% of the participants reached life-space level 5 as LS-M and 27.5% of urban participants reached life-space level 5 independently without any support (LS-I). In contrast, 79.1% of rural participants achieved LS-level 5 as LS-M and 37.2% did this independently without any support (LS-I). Figure 1 illustrates the different life-space measures among the total sample and urban/rural subsample. No significant differences between urban and rural participants were observed in LS-C: (t(81)=1.00; p=.323), LS-E: (t(81)=0.57; p=.571), and LS-D: (t(80.99)=-1.95; p=.054). Rural participants had a significantly higher LS-M: (t(64.60)=3.83; p<.001), and LS-I: (t(77)=-2.00; p=.049).

Construct validity

For the total sample, associations from the bivariate regression analyses between the LSA-D composite score, demographic variables, functional mobility and other health measures were significant for age (β=-.24; 95%CI=-.44/-.07; p=.016), status of shared living arrangements (β=.22; 95%CI=.01/.43; p=.040) ADL/iADL (β=-.23; 95%CI=-.43/-.01; p=.034), TUG (β=-.47; 95%CI=-.66/-.34; p<.001), self-rated health (β=.40; 95%Cl=.19/.61; p<.001), history of falls (β=-.35; 95%CI=-.54/-.15; p<.001). Male gender (β=-.09; 95%CI=-.31/.13; p=.407) and urban residence (β=-.11; 95%CI=-.33/.10; p=.314) were not significant for the total sample. In the adjusted model, age, male gender, urban residence, status of shared living arrangements, number of limitations in ADL/iADL, TUG, self-rated health and history of falls were included into the equation in one step for the total sample. The result revealed significant associations for living status in shared living arrangements (β=.22; 95%CI=.01/.44; p=.045), limitations in ADL/iADL activities (β=-.26; 95%CI=-.42/-.08; p=.008), functional mobility

measured with the TUG (β=-.37; 95%CI=-.68/-.14; p=.008) and history of falls (β=-.22; 95%CI=-.41/-.05; p=.018). No significant associations were found for male gender (β=.15; 95%CI=-.04/.35; p=.135) and urban residence (β=-.19; 95%CI=-.42/.03; p=.090), which corresponds with the bivariate model. However, in contrast to bivariate models, influence of age (β=-.08; 95%CI=-.32/.12; p=.509) and self-rated health (β=.24; 95%CI=.02/.47, p=.058) were not significant in the multivariate model. Results of bivariate and adjusted multivariate regression models are shown in table 2.

Table 2: Unadjusted and adjusted associations of sociodemographic and health factors with the LSA-D composite score (N=83)

Variable	*Bivariate unadjusted models*			*Adjusted model*		
	Beta	*LL CI / UL CI*	*p*	*Beta*	*LL CI / UL CI*	*p*
Age	**-.24**	**-.44/-.07**	**.016**	-.08	-.32/.12	.509
Gender (male)	-.09	-.31/.13	.407	.15	-.04/.35	.135
Living in shared living arrangements	**.22**	**.01/.43**	**.040**	**.22**	**.01/.44**	**.045**
Lives in urban area	-.11	-.33/.10	.314	-.19	-.42/.03	.090
ADL/iADL-Score	**-.23**	**-.43/-.01**	**.034**	**-.26**	**-.42/-.08**	**.008**
TUG	**-.47**	**-.66/-.34**	**< .001**	**-.37**	**-.68/-.14**	**.008**
Self-rated health	**.40**	**.19/.61**	**< .001**	.24	.02/.47	.058
Balance confidence	**.50**	**.33/.67**	**< .001**	-	-	-
History of falls in past 6 months	**-.35**	**-.54/-.15**	**< .001**	**-.22**	**-.41/-.05**	**.018**

Notes: Timed-Up&Go-Test (TUG); Activities of Daily Living (ADL); Instrumental Activities of Daily Living (iADL); p < .05 highlighted in bold

For peer review only

Separate bivariate regression analyses for the urban and rural region demonstrated comparable results in the urban and rural subsample for TUG urban: (β=-.48; 95%CI=-1.14/-.32; p=.008) and rural: (β=-.60; 95%CI=-.95/-.40; p<.001), self-rated health urban: (β=.51; 95%CI=.29/.90; p=.001) and rural: (β=.43; 95%CI=.12/.77; p=.010), balance confidence urban: (β=.67; 95%CI=.38/.93; p=<.001) and rural: (β=.54; 95%CI=.29/.81; p=.001) and history of falls urban: (β=-.31; 95%CI=-.59/-.03; p=.030) and rural: (β=-.41; 95%CI=-.67/-.11; p=.009). Age was significant for those living in the urban region (β=-.31; 95%CL=-.53/-.09; p=.011), but not for the rural sample (β=-.28; 95%CI=-.68/.03; p=.147). All other demographic variables and health measures showed no significant associations in both groups. Results are presented in table 3.

Post-hoc calculations of the adjusted model for each sample separately showed a significant regression coefficient in the urban sample for the score of ADL/iADL limitations (β=-.23; 95%CI=-.41/-.10; p=.035) while coefficients for all other variables were not significant. For the rural population, results for status of shared living arrangements (β=.39; 95%CI=.02/.75; p=.039) and history of falls (β=-.42; 95%CI=-.80/-.08; p=.04) showed significance while other variables did not.

For peer review only

Table 3: Unadjusted associations of sociodemographic and health factors with LSA-D composite score for participants in urban (n=40) and rural areas (n=43)

Variable	*Urban*			*Rural*		
	Beta	*LL CI / UL CI*	*p*	*Beta*	*LL CI / UL CI*	*P*
Age	**-.31**	**-.53/-.09**	**.011**	-.28	-.68/.03	.147
Gender (male)	-.20	-.43/.06	.151	-.04	-.38/.29	.826
Living in shared living arrangements	.18	-.11/.45	.231	.24	-.10/.54	.150
ADL/iADL-Score	-.16	-.39/.11	.209	-.28	-.71/.11	.208
TUG	**-.48**	**-1.14/-.32**	**.008**	**-.60**	**-.95/-.40**	**< .001**
Self-rated health	**.51**	**.29/.90**	**.001**	**.43**	**.12/.77**	**.010**
Balance confidence	**.67**	**.38/.93**	**< .001**	**.54**	**.29/.81**	**.001**
History of falls in past 6 month	**-.31**	**-.59/-.03**	**.030**	**-.41**	**-.67/-.11**	**.009**

Notes: Timed-Up&Go-Test (TUG); Activities of Daily Living (ADL); Instrumental Activities of Daily Living (iADL); p < .05 highlighted in bold

For peer review only

Discussion

We translated and validated a German version of the LSA in urban and rural community-dwelling older adults. In line with the original validation of the LSA[12], moderate associations of the LSA-D composite score with status of living in shared living arrangements, limitations in ADL/iADL as well as with physical performance assessed with the TUG and history of falls were found in the standardized adjusted model. The standardized adjusted association of limitations in ADL/iADL with the LSA-D composite score revealed in our study is in line with findings of Baker et al.[12] and Curcio[22], albeit lower than expected. We found stronger moderate adjusted associations for functional mobility measured with the TUG. These results correspond with findings by Ullrich et al. who reported a moderate Pearson correlation with the TUG when validating the modified LSA-CI to capture life-space of older adults with mild cognitive impairment during the past week.[46] Previous validation studies have tested their version of the LSA composite score against against the Short Physical Performance Battery as a physical assessment of functional mobility and found moderate to strong association.[12, 26] Furthermore, our results revealed a moderate significant association for self-rated health with the LSA-D composite score in bivariate regression, which is in accordance with the original LSA validation study.[12] However, this association did not remain significant in the adjusted model. Unfortunately, balance confidence as an additional subjective health measure that showed moderate significant bivariate associations could not be included in the adjusted model due to multicollinearity.

Our adjusted model confirms the importance of social resources as they can be seen in living together with others in shared living arrangements and functional mobility represented by the significant negative associations with limitations in ADL/iADL activities, time to complete the TUG and a positive history of falls. The importance of social resources was strengthened in post-hoc analyses, where we calculated the adjusted model for each regional

For peer review only

subsample separately. Results showed that living in shared living arrangements and history of falls were significantly associated with life-space mobility in rural areas. This indicates that a nearby social network may play an even more important role in rural areas. In this regard, Kusipar et al.[5] also found evidence for the importance of social support on LS-mobility in the Canadian Longitudinal Study of Aging. Taken together, our findings demonstrate robust evidence for a good construct validity of the LSA-D.

To test our hypothesis that the LSA-D is applicable in both urban and rural living environments, we calculated separate bivariate regressions for each subsample. The associations were similarly strong for functional mobility, self-rated health, balance confidence and history of falls. Although a significant association with age was only found for the urban population and limitations in ADL/iADL were not significant in either subsample, our findings generally correspond across both subsamples. This supports our notion that the LSA-D can be used for measurement of life-space mobility during the past four weeks in community dwelling older adults living in both urban and rural areas.

To further determine construct validity of the LSA-D, we investigated the different scoring methods in the total and both subsamples separately. The LS-M differed between urban and rural participants, with those from rural areas reporting higher LS-M than those living in urban surroundings. Older adults living in rural areas might be more dependent on leaving their village or town in order to gain access to health care services or to run routine errands due to the limited infrastructure often found in rural areas. Our results suggest that the LSA-D is a useful tool for capturing specific characteristics of urban and rural living environments. No group differences were found concerning the LSA-D composite score and the LS-D soring method. This demonstrates the ability of the LSA-D composite score and the dichotomized LS-D score to remain stable and applicable outcome measures in urban and rural living environments.

For peer review only

Strengths and limitations

A main strength of our study is that we tested psychometric properties of the LSA-D among urban and rural community-dwelling older adults. As mobility patterns may vary across living areas, assessments of mobility need to be valid for people living in small villages and large cities as well. Our findings revealed an urban-rural difference in maximal LS and thus demonstrate that the LSA-D can detect disparities in individual mobility patterns that are related to the surrounding living area. These differences must be considered when health care practitioners or researchers address specific questions about independence, social support and functional mobility in different regions. Maximal LS measured with the LS-M score is likely to vary between urban and rural areas and thus may reflect availability of different environmental resources and social support. Another strength is that we applied advanced statistical methods including non-parametric bootstrapping procedures and multivariate regression analysis to account for confounding variables and to estimate the independent association of each of the variables with LSA-D composite-score. However, there are some limitations that need to be considered. First, due to the beginning of the Covid-19 pandemic, we did not reach the planned sample size. However, post-hoc sensitivity analyses revealed a sufficient statistical power. Second, although statistical power was sufficient and the focus was on community-dwelling older adults, our sample size was rather small and non-representative. Future studies should replicate our findings in a representative sample, including subgroups of older adults with mild cognitive impairment, care dependency or living in nursing care arrangements. Moreover, future studies should consider the overlap between LS scoring methods of the LSA-D, social constructs and objectively derived GPS data. This multimodal approach can lead to a better understanding of complex mobility patterns in older adults and associated factors.

For peer review only

Conclusion

In conclusion, the LSA-D has shown good construct validity and can be used in the general population of community-dwelling older adults in urban and rural living environments. The use of LSA-D is recommended for geriatric health care practitioners of different disciplines to assess mobility in the context of social participation and health service utilization.

Acknowledgments

We sincerely thank Patricia Sawyer for her feedback on the pre-final, back translated English LSA-D version. Also, we would like to thank Monica Schanzer and Rudolf Mörgeli for the back translation of the LSA-D, all participants and all members of the study staff that are not listed as authors.

For peer review only

References

1 Webber SC, Porter MM, Menec VH. Mobility in older adults: a comprehensive framework. *Gerontologist* 2010;**50**:443-50.

2 Rantakokko M, Wilkie R. The role of environmental factors for the onset of restricted mobility outside the home among older adults with osteoarthritis: a prospective cohort study. *BMJ Open* 2017;**7**:e012826.

3 Spaltenstein J, Bula C, Santos-Eggimann B, et al. Factors associated with going outdoors frequently: a cross-sectional study among Swiss community-dwelling older adults. *BMJ Open* 2020;**10**:e034248.

4 Giannouli E, Bock O, Mellone S, et al. Mobility in Old Age: Capacity Is Not Performance. *Biomed Res Int* 2016;**2016**:3261567-.

5 Kuspinar A, Verschoor CP, Beauchamp MK, et al. Modifiable factors related to life-space mobility in community-dwelling older adults: results from the Canadian Longitudinal Study on Aging. *BMC Geriatr* 2020;**20**:35.

6 Portegijs E, Keskinen KE, Tsai L-T, et al. Physical Limitations, Walkability, Perceived Environmental Facilitators and Physical Activity of Older Adults in Finland. *International Journal of Environmental Research and Public Health* 2017;**14**:333.

7 May D, Nayak US, Isaacs B. The life-space diary: a measure of mobility in old people at home. *International rehabilitation medicine* 1985;**7**:182-6.

8 Taylor JK, Buchan IE, van der Veer SN. Assessing life-space mobility for a more holistic view on wellbeing in geriatric research and clinical practice. *Aging clinical and experimental research* 2019;**31**:439-45.

9 Hashidate H, Shimada H, Shiomi T, et al. Measuring indoor life-space mobility at home in older adults with difficulty to perform outdoor activities. *J Geriatr Phys Ther* 2013;**36**:109-14.

10 Tinetti ME, Ginter SF. The nursing home life-space diameter. A measure of extent and frequency of mobility among nursing home residents. *Journal of the American Geriatrics Society* 1990;**38**:1311-5.

11 Hauer K, Ullrich P, Heldmann P, et al. Validation of the interview-based life-space assessment in institutionalized settings (LSA-IS) for older persons with and without cognitive impairment. *BMC Geriatrics* 2020;**20**:534.

12 Baker PS, Bodner EV, Allman RM. Measuring life-space mobility in community-dwelling older adults. *Journal of the American Geriatrics Society* 2003;**51**:1610-4.

13 Kennedy RE, Sawyer P, Williams CP, et al. Life-Space Mobility Change Predicts 6-Month Mortality. *Journal of the American Geriatrics Society* 2017;**65**:833-8.

14 Mackey DC, Cauley JA, Barrett-Connor E, et al. Life-space mobility and mortality in older men: a prospective cohort study. *Journal of the American Geriatrics Society* 2014;**62**:1288-96.

15 Silberschmidt S, Kumar A, Raji MM, et al. Life-Space Mobility and Cognitive Decline Among Mexican Americans Aged 75 Years and Older. *Journal of the American Geriatrics Society* 2017;**65**:1514-20.

16 Lo AX, Rundle AG, Buys D, et al. Neighborhood Disadvantage and Life-Space Mobility Are Associated with Incident Falls in Community-Dwelling Older Adults. *Journal of the American Geriatrics Society* 2016;**64**:2218-25.

For peer review only

17 Portegijs E, Rantakokko M, Viljanen A, et al. Is frailty associated with life-space mobility and perceived autonomy in participation outdoors? A longitudinal study. *Age Ageing* 2016;**45**:550-3.

18 Lo AX, Flood KL, Kennedy RE, et al. The Association Between Life-Space and Health Care Utilization in Older Adults with Heart Failure. *J Gerontol A Biol Sci Med Sci* 2015;**70**:1442-7.

19 McCrone A, Smith A, Hooper J, et al. The Life-Space Assessment Measure of Functional Mobility Has Utility in Community-Based Physical Therapist Practice in the United Kingdom. *Physical Therapy* 2019;**99**:1719-31.

20 Sartori AC, Wadley VG, Clay OJ, et al. The relationship between cognitive function and life space: the potential role of personal control beliefs. *Psychology and aging* 2012;**27**:364-74.

21 Saajanaho M, Rantakokko M, Portegijs E, et al. Personal goals and changes in life-space mobility among older people. *Preventive medicine* 2015;**81**:163-7.

22 Curcio CL, Alvarado BE, Gomez F, et al. Life-Space Assessment scale to assess mobility: validation in Latin American older women and men. *Aging clinical and experimental research* 2013;**25**:553-60.

23 Ullrich P, Werner C, Bongartz M, et al. Validation of a Modified Life-Space Assessment in Multimorbid Older Persons With Cognitive Impairment. *Gerontologist* 2019;**59**:e66-e75.

24 Auger C, Demers L, Gelinas I, et al. Development of a French-Canadian version of the Life-Space Assessment (LSA-F): content validity, reliability and applicability for power mobility device users. *Disabil Rehabil Assist Technol* 2009;**4**:31-41.

25 Tseng YC, Gau BS, Lou MF. Validation of the Chinese version of the Life-Space Assessment in community-dwelling older adults. *Geriatr Nurs* 2020;**41**:381-6.

For peer review only

26 Fristedt S, Kammerlind AS, Bravell ME, et al. Concurrent validity of the Swedish version of the life-space assessment questionnaire. *BMC Geriatr* 2016;**16**:181.

27 Pedersen MM, Kjaer-Sorensen P, Midtgaard J, et al. A Danish version of the life-space assessment (LSA-DK) - translation, content validity and cultural adaptation using cognitive interviewing in older mobility limited adults. *BMC Geriatr* 2019;**19**:312.

28 Faul F, Erdfelder E, Buchner A, et al. Statistical power analyses using G*Power 3.1: tests for correlation and regression analyses. *Behavior research methods* 2009;**41**:1149-60.

29 WHO. Process of translation and adaption of instruments. https://www.who.int/substance_abuse/research_tools/translation/en/ 2008.

30 Forsyth BHK, Martha Stapelton; Lawrence, Deidre; Levine, Kerry; Willis Gorgon B. Methods for Translating Survey Questionaires. *American Association for Public Opinion Research* 2006.

31 Ritchie CS, Locher JL, Roth DL, et al. Unintentional weight loss predicts decline in activities of daily living function and life-space mobility over 4 years among community-dwelling older adults. *J Gerontol A Biol Sci Med Sci* 2008;**63**:67-75.

32 Sheppard KD, Sawyer P, Ritchie CS, et al. Life-space mobility predicts nursing home admission over 6 years. *Journal of aging and health* 2013;**25**:907-20.

33 Borsch-Supan A, Brandt M, Hunkler C, et al. Data Resource Profile: the Survey of Health, Ageing and Retirement in Europe (SHARE). *Int J Epidemiol* 2013;**42**:992-1001.

34 Turner G, Clegg A. Best practice guidelines for the management of frailty: a British Geriatrics Society, Age UK and Royal College of General Practitioners report. *Age and Ageing* 2014;**43**:744-7.

35 Podsiadlo D, Richardson S. The timed "Up & Go": a test of basic functional mobility for frail elderly persons. *Journal of the American Geriatrics Society* 1991;**39**:142-8.

For peer review only

36 Lin MR, Hwang HF, Hu MH, et al. Psychometric comparisons of the timed up and go, one-leg stand, functional reach, and Tinetti balance measures in community-dwelling older people. *Journal of the American Geriatrics Society* 2004;**52**:1343-8.

37 Donoghue OA, Savva GM, Cronin H, et al. Using timed up and go and usual gait speed to predict incident disability in daily activities among community-dwelling adults aged 65 and older. *Archives of physical medicine and rehabilitation* 2014;**95**:1954-61.

38 Ludwig K, Graf von der Schulenburg JM, Greiner W. German Value Set for the EQ-5D-5L. *PharmacoEconomics* 2018;**36**:663-74.

39 Schott N. Reliability and validity of the German short version of the Activities specific Balance Confidence (ABC-D6) scale in older adults. *Archives of gerontology and geriatrics* 2014;**59**:272-9.

40 Buchner DM, Hornbrook MC, Kutner NG, et al. Development of the common data base for the FICSIT trials. *Journal of the American Geriatrics Society* 1993;**41**:297-308.

41 Sousa-Santos AR, Amaral TF. Differences in handgrip strength protocols to identify sarcopenia and frailty - a systematic review. *BMC Geriatr* 2017;**17**:238.

42 Charlson ME, Pompei P, Ales KL, et al. A new method of classifying prognostic comorbidity in longitudinal studies: development and validation. *Journal of chronic diseases* 1987;**40**:373-83.

43 Borson S, Scanlan J, Brush M, et al. The mini-cog: a cognitive 'vital signs' measure for dementia screening in multi-lingual elderly. *International journal of geriatric psychiatry* 2000;**15**:1021-7.

44 Hesterberg TMDSM, S. ; Clipson, A. ; Epstein, R. Bootstrap methods and permutation tests. In: A. MDSMGPCB, ed. *Intoduction to practice of statistics*. New York: Palgrave Macmillan 2007.

For peer review only

45 Rasch D, Kubinger KD, Moder K. The two-sample t test: pre-testing its assumptions does not pay off. *Statistical Papers* 2011;**52**:219-31.

46 Ullrich P, Eckert T, Bongartz M, et al. Life-space mobility in older persons with cognitive impairment after discharge from geriatric rehabilitation. *Archives of gerontology and geriatrics* 2019;**81**:192-200.

For peer review only

Footnotes

Contributors: JK developed the first draft of the study. JK, SAM and PG planned and conducted the final studyprotocol. JK and SAM translated LSA into German, compiled the final version of the LSA-D, and contributed in recruiting and testing participants. SAM drafted the manuscript and conducted the major analyses. MS drafted tables and the figure. PG established the statistical analysis plan and performed the sample size and power calculation. MS assisted with data analyses and interpretation of the results. JLOS gave advice and commented on the manuscript. All authors critically reviewed the manuscript and approved the final version.

Funding: The study was partly financed by the "MOBILE"-Project funded by the Federal Ministry of Education and Research (BMBF; ID 01GY1803) and the Institute of Medical Sociology and Rehabilitation Science, Charité – Universitätsmedizin Berlin. We acknowledge support from the German Research Foundation (DFG) and the Open Access Publication Fund of Charité – Universitätsmedizin Berlin.

Competing Interests: Non declared.

Patient consent for publication: Not required

Patient and public involvement: Patients and/or the public were not involved in the design, or conduct, or reporting, or dissemination plans of this research.

Ethical approval: Ethics approval for the study was provided by the Ethics Committee of Charité – Universitätsmedizin Berlin (Rec. Reference EA2/124/19).

Data availability statement: Data will be made available on reasonable, methodologically sound request as soon as possible to achieve the aims of the approved proposal.

Available data includes individual participant data that underlies the results reported in this article after its deidentification, data dictionary and the analytical code.

Data will be available at least 3 months after, and ending 5 years following article publication.

Proposals should be directed at sandra.muemken@charite.de or joern.kiselev@charite.de

For peer review only

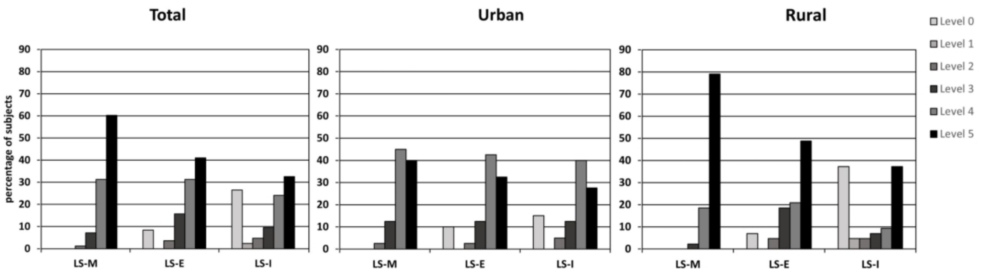

Distribution of different life-space levels of the LSA-D among the total sample, urban and rural subsample. LS-M = maximal life-space; LS-E = life-space with equipment: LS-I = independent life-space; LS-C composite life-space

209x69mm (300 x 300 DPI)

German Life-Space-Assessment LSA-D

DIE NÄCHSTEN FRAGEN BEZIEHEN SICH AUF IHRE AKTIVITÄTEN IN DEN LETZTEN VIER WOCHEN:			A. WIE HÄUFIG WAREN SIE IN DEN LETZTEN 4 WOCHEN IN (Name des Life-Space-Levels)? Häufigkeit				WIE SIND SIE DORT HINGEKOMMEN? B. HABEN SIE HILFSMITTEL ODER ANDERE AUSRÜSTUNG DAFÜR VERWENDET?			C. BENÖTIGTEN SIE DAFÜR DIE HILFE EINER ANDEREN PERSON?		
WAREN SIE IN DEN LETZTEN 4 WOCHEN…	Ja	Nein	Weniger als 1 mal die Woche	1-3 mal die Woche	4-6 mal die Woche	Täglich	Ja	Nein	Unbekannt / keine Angabe	Ja	Nein	Unbekannt / keine Angabe
IN ANDEREN RÄUMEN IHRES ZUHAUSES außer dem Raum, in dem Sie schlafen? *LIFE-SPACE 1*	**LS1** ❑	❑	**LS1-Häufigkeit** ❑	❑	❑	❑	**LS1-Hilfsmittel** ❑	❑	❑	**LS1-Persönliche Hilfe** ❑	❑	❑
IN DER NÄHEREN UMGEBUNG AUSSERHALB IHRER WOHNUNG (Hausflur, Terrasse, Balkon, Fahrstuhl, Hof, Garage, hauseigener Garten, Auffahrt)? *LIFE-SPACE 2*	**LS2** ❑	❑	**LS2-Häufigkeit** ❑	❑	❑	❑	**LS2-Hilfsmittel** ❑	❑	❑	**LS2-Persönliche Hilfe** ❑	❑	❑
AN ORTEN IN IHRER NACHBARSCHAFT, aber außerhalb Ihrer Wohnung oder Ihrer näheren Wohnumgebung? *LIFE-SPACE 3*	**LS3** ❑	❑	**LS3-Häufigkeit** ❑	❑	❑	❑	**LS3-Hilfsmittel** ❑	❑	❑	**LS3-Persönliche Hilfe** ❑	❑	❑
AN ORTEN AUSSERHALB IHRER NACHBARSCHAFT, aber innerhalb der Stadt oder der Ortschaft, in der Sie leben? *LIFE-SPACE 4*	**LS4** ❑	❑	**LS4-Häufigkeit** ❑	❑	❑	❑	**LS4-Hilfsmittel** ❑	❑	❑	**LS4-Persönliche Hilfe** ❑	❑	❑
AN ORTEN AUSSERHALB DER STADT ODER DER ORTSCHAFT, IN DER SIE LEBEN? *LIFE-SPACE 5*	**LS5** ❑	❑	**LS5-Häufigkeit** ❑	❑	❑	❑	**LS5-Hilfsmittel** ❑	❑	❑	**LS5-Persönliche Hilfe** ❑	❑	❑

For peer review only

BMJ Open

Validation of the German Life-Space Assessment LSA-D: Cross sectional validation study in urban and rural community-dwelling older adults

Journal:	*BMJ Open*
Manuscript ID	bmjopen-2021-049926.R1
Article Type:	Original research
Date Submitted by the Author:	02-Jun-2021
Complete List of Authors:	Mümken, Sandra Angelika; Charité Universitätsmedizin Berlin, Institute of Medical Sociology and Rehabilitation Science<br>Gellert, Paul; Charité Universitätsmedizin Berlin, Institute of Medical Sociology and Rehabilitation Science<br>Stollwerck, Malte; Charité Universitätsmedizin Berlin, Institute of Medical Sociology and Rehabilitation Science<br>O'Sullivan, Julie Lorraine; Charité Universitätsmedizin Berlin, Institute of Medical Sociology and Rehabilitation Science<br>Kiselev, Joern; Charité Universitätsmedizin Berlin, Department of Anesthesiology and Operative Intensive Care Medicine (CCM, CVK)
**Primary Subject Heading**:	Rehabilitation medicine
Secondary Subject Heading:	Geriatric medicine, Public health, Diagnostics, Evidence based practice
Keywords:	Rehabilitation medicine < INTERNAL MEDICINE, GERIATRIC MEDICINE, SOCIAL MEDICINE

SCHOLARONE™
Manuscripts

I, the Submitting Author has the right to grant and does grant on behalf of all authors of the Work (as defined in the below author licence), an exclusive licence and/or a non-exclusive licence for contributions from authors who are: i) UK Crown employees; ii) where BMJ has agreed a CC-BY licence shall apply, and/or iii) in accordance with the terms applicable for US Federal Government officers or employees acting as part of their official duties; on a worldwide, perpetual, irrevocable, royalty-free basis to BMJ Publishing Group Ltd ("BMJ") its licensees and where the relevant Journal is co-owned by BMJ to the co-owners of the Journal, to publish the Work in this journal and any other BMJ products and to exploit all rights, as set out in our licence.

The Submitting Author accepts and understands that any supply made under these terms is made by BMJ to the Submitting Author unless you are acting as an employee on behalf of your employer or a postgraduate student of an affiliated institution which is paying any applicable article publishing charge ("APC") for Open Access articles. Where the Submitting Author wishes to make the Work available on an Open Access basis (and intends to pay the relevant APC), the terms of reuse of such Open Access shall be governed by a Creative Commons licence – details of these licences and which Creative Commons licence will apply to this Work are set out in our licence referred to above.

Other than as permitted in any relevant BMJ Author's Self Archiving Policies, I confirm this Work has not been accepted for publication elsewhere, is not being considered for publication elsewhere and does not duplicate material already published. I confirm all authors consent to publication of this Work and authorise the granting of this licence.

For peer review only

Validation of the German Life-Space Assessment LSA-D: Cross sectional validation study in urban and rural community-dwelling older adults

Sandra A. Mümken[1]

Paul Gellert[1]

Malte Stollwerck[1]

Julie L. O'Sullivan[1]

Joern Kiselev[2]

[1] Institute of Medical Sociology and Rehabilitation Science, Charité – Universitätsmedizin Berlin, Germany, corporate member of Freie Universität Berlin, Humboldt-Universität zu Berlin, and Berlin Institute of Health, Charitéplatz 1, D-10117 Berlin, Germany

[2] Department of Anesthesiology and Operative Intensive Care Medicine (CCM, CVK), Charité Universitätsmedizin Berlin, corporate member of Freie Universität Berlin, Humboldt-Universität zu Berlin, and Berlin Institute of Health, Charitéplatz 1, D-10117 Berlin, Germany

Corresponding author:

Sandra A. Mümken

Institute of Medical Sociology and Rehabilitation Science

Charitéplatz 1 10117 Berlin

sandra.muemken@charite.de

Wordcount: 4.264

For peer review only

Abstract

Objectives To develop a German version of the original University of Alabama at Birmingham (UAB) Study of Aging Life-Space Assessment (LSA-D) for measurement of community mobility in older adults within the past 4 weeks and to evaluate its construct validity for urban and rural populations of older adults.

Design Cross-sectional validation study.

Setting Two study centres in urban and rural German outpatient hospital settings.

Participants In total N=83 community-dwelling older adults were recruited (n=40 from urban and n=43 from rural areas; mean age was 78.5 (SD=5.4); 49% male).

Primary and secondary outcome measures The final version of the translated LSA-D was related with limitations in activities and instrumental activities of daily living (ADL/iADL) as primary outcome measure (primary hypothesis) and with sociodemographic factors, functional mobility, self-rated health, balance confidence and history of falls as secondary outcome measures to obtain construct validity. Further descriptive measurements of health included handgrip strength, screening of cognitive function, comorbidities and use of transportation. To assess construct validity, correlations between LSA-D and the primary and secondary outcome measures were examined for the total sample, and urban and rural subsamples using bivariate regression and multiple adjusted regression models. Descriptive analyses of LSA-D included different scoring methods for each region. All parameters were estimated using non-parametric bootstrapping procedure.

Results In the multiple adjusted model for the total sample, number of ADL/iADL limitations (β=-.26; 95%CI=-.42/-.08), TUG (β=-.37; 95%CI=-.68/-.14), shared living arrangements (β=.22; 95%CI=.01/.44) and history of falls in the past 6 months (β=-.22; 95%CI=-.41/-.05) showed significant associations with the LSA-D composite score, while living in urban area (β=-.19; 95%CI=-.42/.03) and male gender (β=.15; 95%CI=-.04/.35) were not significant.

For peer review only

Conclusion The LSA-D is a valid tool for measuring life-space mobility in German community-dwelling older adults within the past four weeks in ambulant urban and rural settings.

Trial registration number DRKS00019023

Keywords: Older adults; Mobility; Life-space; German; Validation; living environment; geriatrics,

Strengths and limitations of this study

- German validation of the original UAB Life-Space Assessment (LSA-D) for community dwelling older adults in urban and rural settings
- Using bootstrapped bivariate and multiple adjusted regression models to attain construct validity of the LSA-D
- Recruitment had to be stopped shortly before reaching the calculated sample size due to the decision to restrict in face-to-face research to contain the outbreak of the Covid-19 pandemic in March 2020

For peer review only

Introduction

Mobility, defined as “the ability to move oneself (either independently or by using assistive devices or transportation) within environments that expand from one’s home to the neighbourhood and regions beyond”[1] encompasses general independence, opportunities for social activities and freedom to experience new sites. This broad concept of mobility goes beyond the narrow conception of mobility as performance in a single functional test without considering environmental barriers and social resources although their impact on mobility has been investigated.[2, 3] Therefore, the focus on single functional mobility tests can lead to misconceptions about actual mobility performance in everyday life and health practitioners may oversee possible consequences for social participation and mental health.[4]

To overcome these shortcomings of functional mobility assessments, recent studies of mobility and aging operationalize mobility as circled areas, so-called life-spaces, that spread from the centre of one’s own house and garden to the neighbourhood, the city lived in and beyond, with each life-space offering different opportunities for social involvement, recreational activities or access to medical care.[5, 6] The application of self-reported life-spaces to determine mobility of older adults was first established by May et al. in 1985[7] and assessment of life-space mobility with standardized questionnaires was recently recommended for geriatric research.[8] Several instruments for measuring life-space mobility in specific populations and settings exist, including assessments of life-space within one’s own residence for home-bound individuals[9] or residents in nursing homes and other institutions.[10, 11]

One of the most frequently applied instruments for measurement of mobility in older adults using the life-space concept is the validated Life-Space Assessment (LSA) by Baker et al.[12] as part of the University of Alabama at Birmingham (UAB). Study of Aging. The LSA provides health professionals in geriatric settings with information on availability of environmental and

For peer review only

social resources as an outcome of mobility assessment and gives them a more comprehensive picture of the patient's needs.

The importance of the LSA for clinical practice has been shown in various studies. Kennedy et al.[13] for instance found that a decline in life-space mobility over six months is associated with greater mortality in the following six months. Limitations in life-space mobility are associated with long-term mortality of older men[14], cognitive decline[15], fall risk [16], frailty[17] and hospital admission in older adults with heart failure.[18] Furthermore, the concept has already been established in outpatient physical therapy with various community-dwelling neurological orthopaedic and surgery patients.[19] Additionally, psychological health factors like external control beliefs [20] and personal activity goals [21] influence life-space mobility. Therefore, the LSA can also supplement evaluation concepts in psychological research and treatment of older adults. The construct validity of the LSA was commonly tested by relating the LSA to activities and instrumental activities of daily living (ADL/iADL) but also self-rated health and fears of falling.[22, 23, 24] Moreover, as pointed out by Baker et al.[12], there is a need to validate the LSA for urban and rural settings. Recently published studies also indicate environmental factors, such as distance to services or quality of streets and sidewalks, that differ between urban and rural settings might influence life-space mobility by reducing or maximising the opportunities to move independently outdoors and participate in social activities.[25]

As part of validity testing, the LSA has been translated into multiple languages such as Chinese[26], French[24], Spanish[22], Swedish[27] or Danish[28]. To date, two modified German versions for assessment of life-space mobility in specific populations of older adults exist: the LSA-CI captures life-space mobility of the past week for those with mild cognitive impairment [23]. In comparison, the LSA-IS is used in institutionalized settings where life-spaces are adapted to the living environment of care facilities and life-space mobility of the previous day is captured.[11, 29] However, a validated and intercultural adapted version of the original LSA that can be administered in the context of a more general geriatric setting in

For peer review only

the overall population of community-dwelling older adults is still missing. Therefore, we conducted a validation study of a German version of the original LSA (LSA-D) (see online supplement S1.) in urban and rural areas.

Aims and hypotheses

Our aim was to translate, apply and validate the LSA-D, a German version of the LSA from the UAB Study of Aging for the population of urban and rural community-dwelling older adults. In line with the original validation of the LSA we expected a moderate association of the LSA-D composite score with limitations in ADL/iADL as primary hypothesis.[12] As secondary hypothesizes, we assumed moderate associations with sociodemographic measures[12, 22], functional mobility[23, 30] self-rated health[12, 22], balance confidence and history of falls[16, 31]. In a further step we investigated the independent predictive validity of the proposed factors (limitations in ADL/iADL, sociodemographic measures, functional mobility, self-rated health, balance confidence and history of falls) assuming that the primary correlation of limitations in ADL/iADL is present even after adjustment for the other constructs. Finally, we expected the newly translated LSA-D to show patterns of similar strong associations in the urban and rural subsample.

Methods

Study design

A cross-sectional study design was used with two German hospital clinics as study centres. The first study centre was an ambulant geriatric rehabilitation facility of the Havelland clinics located in a small town (16,000 inhabitants) in Brandenburg, Germany. The second centre was based at the Charité – Universitätsmedizin Berlin within the Department of Anesthesiology and Operative Intensive Care. Approvement for the study was given by the local Ethics Committee of the Charité – Universitätsmedizin Berlin (EA2/124/19) and the study was prospectively registered at the German Clinical Trials Register (DRKS00019023).

For peer review only - http://bmjopen.bmj.com/site/about/guidelines.xhtml

Sample size calculation was based on assumptions to find a moderate-to-strong association of beta/r=-.40[12] between the LSA-D-composite score and limitations in ADL/iADL (i.e., primary hypothesis), sociodemographic measures, functional mobility, self-rated health and balance confidence in all observed populations. For testing of the primary hypothesis, 92 participants or 46 subjects per setting (i.e., urban/rural) were required. This was based on the following assumptions: An effect size of Pearson's correlation coefficient or standardized beta coefficient of r/beta=-0.40 (ρ=-0.40 in the population) was assumed in reference to the association between the LSA composite score (LS-C) and limitations in ADL/iADL found in the original validation study of LSA.[12] The power calculation with GPower 3.1 for bivariate correlations (test family "exact")[32] resulted in an estimated minimum sample size of n=46 participants per setting (urban/rural) and a critical r=-0.29 with a type I error rate of alpha=0.025 (test one-sided; corrected for multiple testing [setting urban/rural; alpha=0.05/2]) and a statistical power of 1-β=0.80. Recruitment commenced in November 2019 and had to be stopped in March 2020 at a sample size of 82 due to restrictions of the then starting coronavirus pandemic. A post hoc sensitivity analysis suggests that we are still able to detect effects of r=-.30 and larger.

Translation process

In accordance with the 2008 guidelines of the World Health Organization[33], forward translation into German language was separately conducted by two researchers who formulated two German versions that were discussed and then merged into one German pre-version of the LSA-D. The pre-version was given to two native English speakers for back translation. Again, both versions of the back translation were discussed by the two native speakers and a concerted version of the back-translation was produced. Differences between the original LSA and the concerted back-translation were discussed and reviewed with the original author of the LSA to redefine a pre-final version of the LSA-D that was pre-tested for understandability using

For peer review only

cognitive interview technique among 4 older adults of the Charité – Universitätsmedizin Berlin to create the final LSA-D version.[34]

Participants and recruitment

The 83 participants were divided into two groups mainly based on the size of their place of residence and taking Chistaller's theory of "central places" into consideration that categorizes living areas based on provided services and infrastructure.[35] Participants from villages (up to 5,000 inhabitants) and small towns (up to 40,000 inhabitants) were classified as living in rural areas as some towns missed to provide services of upscale daily needs (e.g. public swimming pools). In contrast, participants who lived in the city of Berlin (3.8 million inhabitants) with its metropolitan infrastructure and services were classified as urban population.

Inclusion criteria were defined as: age of 70 years and older; ability to read and understand the questionnaire and give written informed consent. Exclusion criteria were incidences that limited mobility within the past four weeks, known diagnosed severe cognitive limitations or mental conditions, need of acute care and insufficient understanding of the German language. In total, 126 persons were screened for eligibility of which 28 did not fulfil the inclusion criteria and 15 were unwilling to participate. In both study centres, participants were recruited during normal health care routine by trained study staff and medical professionals were consulted by any uncertainty regarding the participant´s eligibility. All participants received verbal and written information on the study and were given time to consider participation before giving written consent.

For peer review only - http://bmjopen.bmj.com/site/about/guidelines.xhtml

Measures

Selection of primary and secondary variables for determining construct validity was based on the original validation study of the UAB and other LSA validation studies from different countries. [12, 22, 23, 31]

Primary outcome measures

Life-space mobility was evaluated with the translated German Version of the UAB Life-Space Assessment. The LSA consists of a questionnaire on five different life-spaces capturing six possible levels of life-space (0. mobility within the bedroom; 1. rooms inside the home besides the bedroom; 2. area outside the house; 3. neighbourhood; 4. town or city lived in; 5. outside of town or city lived in). For each level, participants were asked a) if they went to this level in the past four weeks, b) if so, how often, c) if they needed assistive devices or special equipment to reach that level and d) if they needed personal help to reach that level.[12] Different scoring methods can be used with the LSA either indicating the maximum attained life-space level (LS-M), life-space that can be reached independently without any further support (LS-I), reachable life-space with possible use of equipment but without personal help (LS-E), dichotomized life-space (LS-D) that classifies a person's mobility into the ability to travel beyond the borders of their self-perceived neighbourhood and the composite score (LS-C) that summarizes the attained LS-level, needed equipment or personal support and frequency of visits. The LS-C score ranges from 0 to 120 points with higher scores indicating better mobility. As the LS-C score has shown a good sensitivity regarding change over time, it is frequently applied in longitudinal studies.[36, 37] In cross-sectional studies, LS-I and LS-D are additional scores for describing actual mobility and associations with other health factors.[12]

Limitations in ADL/iADL were investigated using questions from the "Survey of Aging and Retirement in Europe" (SHARE).[38] Participants were asked whether they had difficulties due to physical, emotional or cognitive problems to perform 15 activities like dressing,

For peer review only

gardening, using a map or making a telephone call. Binary response options for each activity were yes or no. Subsequently, a sum score of limitations in ADL/iADL activities was calculated ranging from 0 to 15. Higher scores indicate more functional impairments.

Secondary outcome measures

Sociodemographic factors (i.e., age, gender, height, weight, status of shared living-arrangements) use of public transportation and driving-status were assessed with a standardized questionnaire.

The "Timed-Up&-Go-Test" TUG is one of the most frequently used measures of balance and functional mobility in older adults and is a recommended tool for geriatric assessment.[39] During performance of the TUG, time (in seconds) is taken for rising up from a standardized chair, walking three metres, turning around, walking back and siting down again at a comfortable self-selected speed.[40] Higher TUG times are associated with impaired mobility.[41, 42]

The EQ-VAS from the EQ-5D-5L version was used to record overall self-rated health of the day on a vertical visual analogue scale ranging from 0 points for the worst imaginable health to 100 points for the best conceivable health.[43] To measure balance confidence, we used the ABC-6-Scale that was translated into German and validated by Schott et al.[44] Participants were accounted to have a history of falls if they had fallen at least one time in the past six months using the criteria of the "Frailty and Injuries: Cooperative Studies of Intervention Techniques" to define a fall.[45]

Further descriptive measures of health

Hand grip strength was measured as maximum of three contractions with a hydraulic handheld dynamometer (Sahean SH5001; Changwon, South Korea) in the dominant hand and standardized sitting position.[46] We administered the Charlson comorbidity index (CCI) as a method to categorize comorbidities (0-41 points) where scores of >5 indicate a higher mortality risk.[47] Cognitive status was assessed with the Mini-Cog screening tool where a score ranging

For peer review only

from 0-5 can be achieved and a score of 0-2 is seen as an indicator for further investigation of cognitive status.[48]

Statistical analysis

Means (M) and standard deviations (SD) were reported descriptively for continuous demographic variables (i.e., age, height, weight) and health measures (i.e., limitations in ADL/iADL, time in seconds needed to complete TUG, self-rated health and balance confidence). Gender, status of shared living arrangements, use of different transportation modes and history of falls were reported for the total and each subsample as absolute frequencies and percentage of participants. Distribution of the data was skewed therefore we used the non-parametric, bias corrected and accelerated (BCA) bootstrap method with 10,000 resamples and fixed random seeds that resamples the collected data with replacement to derive robust results.[49] With the BCA bootstrap method, coefficients and confidence intervals can be estimated with good statistical power even if sample sizes are small and distribution of data is unknown or not normal. For investigating differences between urban and rural participants, the Welch Test was performed as it has been recommended as a standard test for small samples.[50] To determine construct validity of the LSA-D, BCA bootstrap method and standardized z-scores (i.e., that can be interpreted like beta coefficients) of the included binary and continuous variables (i.e., age, male gender, rural or urban residence, status of shared living arrangements, sum score of limitations with ADL/iADL activities, functional mobility with TUG, self-rated health and history of falls) were used for bivariate and multivariate regression analysis. Balance confidence (scores of the ABC-6 scale) had to be excluded from multivariate regression because they revealed a correlation of r=-.72 with TUG scores. To avoid multicollinearity, it was decided to include only the TUG score due to its importance as a physical measurement of functional mobility for assessing construct validity. All analyses were run using SPSS version 25. Microsoft Excel 2016 was used to create the figure.

For peer review only - http://bmjopen.bmj.com/site/about/guidelines.xhtml

Results

Sample characteristics

For the total sample (N=83), mean age was 78.5 (SD=5.4) years and about half of the sample (n=41; 49.4%) were male. 47 participants (56.6%) lived together with others in a shared living arrangement. In the past four weeks, 39 participants (47.0%) drove a car by themselves, 18 participants (21.7%) rode a bicycle and 34 participants (41.0%) used walking aids. On average, participants had a TUG of M=13.90 (SD=9.20) seconds. Score of limitations in ADL/iADL was moderate with M=7.8 (SD=6.2) and mean score of self-rated health was M=64.7 (SD=21.3).

When comparing urban with rural participants, those living in urban areas had significantly more ADL/iADL limitations, t(74.51)=-2.34; p=.022, and comorbidities, t(57.27)=-2.44; p=.018. Rural participants were significantly older, t(81)=2.43; p=.017, needed more time to complete the TUG, t(70.65)=3.33; p=.001, had less balance confidence, t(80.11)=-2.84; p=.006 and had lower self-rated health, t(81)=-2.45; p=.016. Concerning the utilization of means of transportation, the percentage of participants who drove a car or a bicycle for independent mobility within the last 4 weeks did not differ significantly across regions. Characteristics of participants in total and separately for each region are presented in table 1.

For peer review only

Table 1: Participant Characteristics

Variable	total (N=83)		urban (*n*=40)		rural (*n*=43)	
	N	*%*	*N*	*%*	*N*	*%*
Gender (male)	41	49.4	23	57.5	18	41.9
Status of shared living arrangements	47	56.6	19	47.5	28	65.1
Drove a car in past 4 weeks	39	47.0	18	45.0	21	48.8
Rode a bicycle in past 4 weeks	18	21.7	9	22.5	9	20.9
Used walking aid in past 4 weeks	34	41.0	11	27.5	23	53.5
History of falls past 6 months (>1)	22	26.5	9	22.5	13	30.2
	M	*SD*	*M*	*SD*	*M*	*SD*
Age (years)	78.5	5.4	77.1	5.2	**79.8***	5.2
Height (cm)	168.7 (3)	10.5	170.8	11.2	166.5 (3)	9.3
Weight (kg)	79.0 (3)	18.8	79.7	19.8	78.3 (3)	17.8
Body Mass Index	27.6 (3)	5.4	27.1	5.8	28.1 (3)	5.1
Charlson Comorbidity Index (0-41)	3.2	3.6	**4.2***	4.5	2.3	2.3
Hand grip strength (kg)	25.8 (2)	11.6	27.7 (1)	11.8	24.0 (1)	11.4
Mini-Cog™-Score (0-5)	3.8	1.5	3.8	1.6	3.8	1.4
LSA-D composite score (0-120)	60.8	24.3	58.0	21.7	63.3	26.5
ADL/iADL (number of limitations; 0-15)	7.8	6.2	**9.4***	6.7	6.2	5.4
TUG (s)	13.9 (5)	9.2	10.5 (3)	6.8	**16.9**(2)**	10.1
Self-rated health (0-100)	64.8	21.3	70.5	19.3	**59.4***	21.8
Balance confidence (0-100)	57.8	32.7	67.8	28.4	**48.4***	34.0

Note: Live-Space-Assessment-Deutsch (LSA-D); Activities of Daily Living (ADL); Instrumented Activities of Daily Living (iADL); Timed-Up &Go-Test (TUG); Numbers in brackets report number of missing values;
* significant difference between subsamples (p<.05)
** significant difference between subsamples (p<.001)

For peer review only

Descriptive statistics of the LSA-D

Life-space-level 5, as maximum life-space (LS-M), was reached by 60.2% of the total sample. 32.5% of all participants had an independent life-space level (LS-I) of 5 while the remaining needed either equipment or personal help. For the urban subsample, 40.0% of the participants reached life-space level 5 as LS-M and 27.5% of urban participants reached life-space level 5 independently without any support (LS-I). In contrast, 79.1% of rural participants achieved LS-level 5 as LS-M and 37.2% did this independently without any support (LS-I). Figure 1 illustrates the different life-space measures among the total sample and urban/rural subsample. No significant differences between urban and rural participants were observed in LS-C: ($t(81)=1.00$; $p=.323$), LS-E: ($t(81)=0.57$; $p=.571$), and LS-D: ($t(80.99)=-1.95$; $p=.054$). Rural participants had a significantly higher LS-M: ($t(64.60)=3.83$; $p<.001$), and LS-I: ($t(77)=-2.00$; $p=.049$).

Construct validity

For the total sample, associations from the bivariate regression analyses between the LSA-D composite score, demographic variables, functional mobility and other health measures were significant for age ($\beta=-.24$; 95%CI=-.44/-.07; $p=.016$), status of shared living arrangements ($\beta=.22$; 95%CI=.01/.43; $p=.040$) ADL/iADL ($\beta=-.23$; 95%CI=-.43/-.01; $p=.034$), TUG ($\beta=-.47$; 95%CI=-.66/-.34; $p<.001$), self-rated health ($\beta=.40$; 95%Cl=.19/.61; $p<.001$), history of falls ($\beta=-.35$; 95%CI=-.54/-.15; $p<.001$). Male gender ($\beta=-.09$; 95%CI=-.31/.13; $p=.407$) and urban residence ($\beta=-.11$; 95%CI=-.33/.10; $p=.314$) were not significant for the total sample. In the adjusted model, age, male gender, urban residence, status of shared living arrangements, number of limitations in ADL/iADL, TUG, self-rated health and history of falls were included into the equation in one step for the total sample. The result revealed significant associations for living status in shared living arrangements ($\beta=.22$; 95%CI=.01/.44; $p=.045$), limitations in ADL/iADL activities ($\beta=-.26$; 95%CI=-.42/-.08; $p=.008$), functional mobility

measured with the TUG (β=-.37; 95%CI=-.68/-.14; p=.008) and history of falls (β=-.22; 95%CI=-.41/-.05; p=.018). No significant associations were found for male gender (β=.15; 95%CI=-.04/.35; p=.135) and urban residence (β=-.19; 95%CI=-.42/.03; p=.090), which corresponds with the bivariate model. However, in contrast to bivariate models, influence of age (β=-.08; 95%CI=-.32/.12; p=.509) and self-rated health (β=.24; 95%CI=.02/.47, p=.058) were not significant in the multivariate model. Results of bivariate and adjusted multivariate regression models are shown in table 2.

Table 2: Unadjusted and adjusted associations of sociodemographic and health factors with the LSA-D composite score (N=83)

Variable	*Bivariate unadjusted models*			*Adjusted model*		
	Beta	*LL CI / UL CI*	*p*	*Beta*	*LL CI / UL CI*	*p*
Age	**-.24**	**-.44/-.07**	**.016**	-.08	-.32/.12	.509
Gender (male)	-.09	-.31/.13	.407	.15	-.04/.35	.135
Status of shared living arrangements	**.22**	**.01/.43**	**.040**	**.22**	**.01/.44**	**.045**
Lives in urban area	-.11	-.33/.10	.314	-.19	-.42/.03	.090
ADL/iADL-Score	**-.23**	**-.43/-.01**	**.034**	**-.26**	**-.42/-.08**	**.008**
TUG	**-.47**	**-.66/-.34**	**< .001**	**-.37**	**-.68/-.14**	**.008**
Self-rated health	**.40**	**.19/.61**	**< .001**	.24	.02/.47	.058
Balance confidence	**.50**	**.33/.67**	**< .001**	-	-	-
History of falls in past 6 months	**-.35**	**-.54/-.15**	**< .001**	**-.22**	**-.41/-.05**	**.018**

Notes: Timed-Up&Go-Test (TUG); Activities of Daily Living (ADL); Instrumental Activities of Daily Living (iADL); p < .05 highlighted in bold

For peer review only

Separate bivariate regression analyses for the urban and rural region demonstrated comparable results in the urban and rural subsample for TUG urban: (β=-.48; 95%CI=-1.14/-.32; p=.008) and rural: (β=-.60; 95%CI=-.95/-.40; p<.001), self-rated health urban: (β=.51; 95%CI=.29/.90; p=.001) and rural: (β=.43; 95%CI=.12/.77; p=.010), balance confidence urban: (β=.67; 95%CI=.38/.93; p=<.001) and rural: (β=.54; 95%CI=.29/.81; p=.001) and history of falls urban: (β=-.31; 95%CI=-.59/-.03; p=.030) and rural: (β=-.41; 95%CI=-.67/-.11; p=.009). Age was significant for those living in the urban region (β=-.31; 95%CL=-.53/-.09; p=.011), but not for the rural sample (β=-.28; 95%CI=-.68/.03; p=.147). All other demographic variables and health measures showed no significant associations in both groups. Results are presented in table 3.

Calculations of the adjusted model for each subsample separately showed a significant regression coefficient in the urban sample for the score of ADL/iADL limitations (β=-.23; 95%CI=-.41/-.10; p=.035) while coefficients for all other variables were not significant. For the rural population, results for status of shared living arrangements (β=.39; 95%CI=.02/.75; p=.039) and history of falls (β=-.42; 95%CI=-.80/-.08; p=.04) showed significance while other variables did not.

For peer review only

Table 3: Unadjusted associations of sociodemographic and health factors with LSA-D composite score for participants in urban (n=40) and rural areas (n=43)

Variable	*Urban*			*Rural*		
	Beta	*LL CI / UL CI*	*p*	*Beta*	*LL CI / UL CI*	*P*
Age	**-.31**	**-.53/-.09**	**.011**	-.28	-.68/.03	.147
Gender (male)	-.20	-.43/.06	.151	-.04	-.38/.29	.826
Status of shared living arrangements	.18	-.11/.45	.231	.24	-.10/.54	.150
ADL/iADL-Score	-.16	-.39/.11	.209	-.28	-.71/.11	.208
TUG	**-.48**	**-1.14/-.32**	**.008**	**-.60**	**-.95/-.40**	**< .001**
Self-rated health	**.51**	**.29/.90**	**.001**	**.43**	**.12/.77**	**.010**
Balance confidence	**.67**	**.38/.93**	**< .001**	**.54**	**.29/.81**	**.001**
History of falls in past 6 month	**-.31**	**-.59/-.03**	**.030**	**-.41**	**-.67/-.11**	**.009**

Notes: Timed-Up&Go-Test (TUG); Activities of Daily Living (ADL); Instrumental Activities of Daily Living (iADL); p < .05 highlighted in bold

For peer review only

Discussion

We translated and validated a German version of the LSA in urban and rural community-dwelling older adults. In line with the original validation of the LSA[12], moderate associations of the LSA-D composite score with limitations in ADL/iADL, living in shared living arrangements as well as with functional mobility assessed with the TUG and history of falls were found in the bivariate regression and standardized adjusted model. The standardized adjusted association of limitations in ADL/iADL with the LSA-D composite score revealed in our study is in line with findings of Baker et al.[12] and Curcio[22], albeit lower than expected. We found stronger moderate adjusted associations for functional mobility measured with the TUG. These results correspond with findings by Ullrich et al.[23] who reported a moderate Pearson correlation with the TUG when validating the modified LSA-CI to capture life-space of older adults with mild cognitive impairment during the past week.[23, 30] Previous validation studies have tested their version of the LSA composite score against against the Short Physical Performance Battery as a physical assessment of functional mobility and found moderate to strong association.[12, 27] Furthermore, our results revealed a moderate significant association for self-rated health with the LSA-D composite score in bivariate regression, which is in accordance with the original LSA validation study.[12] However, this association did not remain significant in the adjusted model. Unfortunately, balance confidence as an additional subjective health measure that showed moderate significant bivariate associations could not be included in the adjusted model due to multicollinearity. Our adjusted model confirms the importance of social resources as they can be seen in living together with others in shared living arrangements and functional mobility represented by the significant negative associations with limitations in ADL/iADL activities, time to complete the TUG and a positive history of falls.

To test our hypothesis that the LSA-D is applicable in both urban and rural living environments, we calculated separate bivariate regressions for each subsample. The associations were

For peer review only

similarly strong for functional mobility, self-rated health, balance confidence and history of falls. Although a significant association with age was only found for the urban population and limitations in ADL/iADL were not significant in either subsample, our findings generally correspond across both subsamples. This supports our notion that the LSA-D can be used for measurement of life-space mobility during the past four weeks in community dwelling older adults living in both urban and rural areas.

Contrary to our expectation results of the adjusted model calculated separately for each subsample revealed that limitations in ADL/iADL were only significantly associated with life-space mobility in urban areas. In contrast, shared living arrangements and history of falls were the only significant adjusted factors in rural areas. One possible explanation could be that life–space mobility achieved on one's own ability's is easier to maintain in urban areas with a more pronounced infrastructure. On the contrary a nearby social network may play a more important role for sustaining life–space mobility in rural areas where distances to services and social activities might be longer. This strengthened the importance of social resources on life-space mobility in rural areas. In this regard, Kusipar et al.[5] also found evidence for the importance of social support on life–space mobility in the Canadian Longitudinal Study of Aging. Due to the small sample size our results should be interpreted with caution and additional studies are needed to confirm he observed differences between urban and rural community-dwelling older adults found in our study. Thereby, future studies should continue to establish a theoretical and empirical basis for urban/rural life-space mobility.

To further determine construct validity of the LSA-D, we investigated the different scoring methods in the total and both subsamples separately. The LS-M differed between urban and rural participants, with those from rural areas reporting higher LS-M than those living in urban surroundings. Older adults living in rural areas might be more dependent on leaving their village or town in order to gain access to health care services or to run routine errands due to

For peer review only - http://bmjopen.bmj.com/site/about/guidelines.xhtml

the limited infrastructure often found in rural areas. Our results suggest that the LSA-D is a useful tool for capturing specific characteristics of urban and rural living environments. No group differences were found concerning the LSA-D composite score and the dichotomized LS-D soring method. This demonstrates the ability of the LSA-D composite score and the dichotomized LS-D score to remain stable and applicable outcome measures in urban and rural living environments. Taken together, our findings demonstrate robust evidence for a good construct validity of the LSA-D.

Strengths and limitations

A main strength of our study is that we tested construct validity of the LSA-D among urban and rural community-dwelling older adults. As mobility patterns may vary across living areas, assessments of mobility need to be valid for people living in small villages and large cities as well. Our findings revealed an urban-rural difference in maximal LS and thus demonstrate that the LSA-D can detect disparities in individual mobility patterns that are related to the surrounding living area. These differences must be considered when health care practitioners or researchers address specific questions about independence, social support and functional mobility in different regions. Maximal LS measured with the LS-M score is likely to vary between urban and rural areas and thus may reflect availability of different environmental resources and social support. Another strength is that we applied advanced statistical methods including non-parametric bootstrapping procedures and multivariate regression analysis to account for confounding variables and to estimate the independent association of each of the variables with LSA-D composite-score. However, there are some limitations that need to be considered. First, due to the beginning of the Covid-19 pandemic, we did not reach the planned sample size. However, post-hoc sensitivity analyses revealed a sufficient statistical power. Second, although statistical power was sufficient and the focus was

For peer review only

on community-dwelling older adults, our sample size was rather small and non-representative. Future studies should replicate our findings in a representative sample, including different subgroups of older adults and evaluate additional psychometric properties of the LSA-D as test-retest reliability and responsiveness. Moreover, future studies should consider the overlap between LS scoring methods of the LSA-D, social constructs and objectively derived GPS data. This multimodal approach can lead to a better understanding of complex mobility patterns in older adults and associated factors.

Conclusion

In conclusion, the LSA-D has shown good construct validity and can be used in the general population of community-dwelling older adults in urban and rural living environments. The use of LSA-D is recommended for geriatric health care practitioners of different disciplines to assess mobility in the context of social participation and health service utilization.

Acknowledgments

We sincerely thank Patricia Sawyer for her feedback on the pre-final, back translated English LSA-D version. Also, we would like to thank Monica Schanzer and Rudolf Mörgeli for the back translation of the LSA-D, all participants and all members of the study staff that are not listed as authors.

Figure 1: Distribution of different life-space levels of the LSA-D among the total sample, urban and rural subsample. LS-M = maximal life-space; LS-E = life-space with equipment: LS-I = independent life-space

For peer review only

References

1 Webber SC, Porter MM, Menec VH. Mobility in older adults: a comprehensive framework. *Gerontologist* 2010;**50**:443-50.

2 Rantakokko M, Wilkie R. The role of environmental factors for the onset of restricted mobility outside the home among older adults with osteoarthritis: a prospective cohort study. *BMJ Open* 2017;**7**:e012826.

3 Spaltenstein J, Bula C, Santos-Eggimann B, et al. Factors associated with going outdoors frequently: a cross-sectional study among Swiss community-dwelling older adults. *BMJ Open* 2020;**10**:e034248.

4 Giannouli E, Bock O, Mellone S, et al. Mobility in Old Age: Capacity Is Not Performance. *Biomed Res Int* 2016;**2016**:3261567-.

5 Kuspinar A, Verschoor CP, Beauchamp MK, et al. Modifiable factors related to life-space mobility in community-dwelling older adults: results from the Canadian Longitudinal Study on Aging. *BMC Geriatr* 2020;**20**:35.

6 Portegijs E, Keskinen KE, Tsai L-T, et al. Physical Limitations, Walkability, Perceived Environmental Facilitators and Physical Activity of Older Adults in Finland. *International Journal of Environmental Research and Public Health* 2017;**14**:333.

7 May D, Nayak US, Isaacs B. The life-space diary: a measure of mobility in old people at home. *International rehabilitation medicine* 1985;**7**:182-6.

8 Taylor JK, Buchan IE, van der Veer SN. Assessing life-space mobility for a more holistic view on wellbeing in geriatric research and clinical practice. *Aging clinical and experimental research* 2019;**31**:439-45.

9 Hashidate H, Shimada H, Shiomi T, et al. Measuring indoor life-space mobility at home in older adults with difficulty to perform outdoor activities. *J Geriatr Phys Ther* 2013;**36**:109-14.

For peer review only

10 Tinetti ME, Ginter SF. The nursing home life-space diameter. A measure of extent and frequency of mobility among nursing home residents. *Journal of the American Geriatrics Society* 1990;**38**:1311-5.

11 Hauer K, Ullrich P, Heldmann P, et al. Validation of the interview-based life-space assessment in institutionalized settings (LSA-IS) for older persons with and without cognitive impairment. *BMC Geriatrics* 2020;**20**:534.

12 Baker PS, Bodner EV, Allman RM. Measuring life-space mobility in community-dwelling older adults. *Journal of the American Geriatrics Society* 2003;**51**:1610-4.

13 Kennedy RE, Sawyer P, Williams CP, et al. Life-Space Mobility Change Predicts 6-Month Mortality. *Journal of the American Geriatrics Society* 2017;**65**:833-8.

14 Mackey DC, Cauley JA, Barrett-Connor E, et al. Life-space mobility and mortality in older men: a prospective cohort study. *Journal of the American Geriatrics Society* 2014;**62**:1288-96.

15 Silberschmidt S, Kumar A, Raji MM, et al. Life-Space Mobility and Cognitive Decline Among Mexican Americans Aged 75 Years and Older. *Journal of the American Geriatrics Society* 2017;**65**:1514-20.

16 Lo AX, Rundle AG, Buys D, et al. Neighborhood Disadvantage and Life-Space Mobility Are Associated with Incident Falls in Community-Dwelling Older Adults. *Journal of the American Geriatrics Society* 2016;**64**:2218-25.

17 Portegijs E, Rantakokko M, Viljanen A, et al. Is frailty associated with life-space mobility and perceived autonomy in participation outdoors? A longitudinal study. *Age Ageing* 2016;**45**:550-3.

18 Lo AX, Flood KL, Kennedy RE, et al. The Association Between Life-Space and Health Care Utilization in Older Adults with Heart Failure. *J Gerontol A Biol Sci Med Sci* 2015;**70**:1442-7.

For peer review only

19 McCrone A, Smith A, Hooper J, et al. The Life-Space Assessment Measure of Functional Mobility Has Utility in Community-Based Physical Therapist Practice in the United Kingdom. *Physical Therapy* 2019;**99**:1719-31.

20 Sartori AC, Wadley VG, Clay OJ, et al. The relationship between cognitive function and life space: the potential role of personal control beliefs. *Psychology and aging* 2012;**27**:364-74.

21 Saajanaho M, Rantakokko M, Portegijs E, et al. Personal goals and changes in life-space mobility among older people. *Preventive medicine* 2015;**81**:163-7.

22 Curcio CL, Alvarado BE, Gomez F, et al. Life-Space Assessment scale to assess mobility: validation in Latin American older women and men. *Aging clinical and experimental research* 2013;**25**:553-60.

23 Ullrich P, Werner C, Bongartz M, et al. Validation of a Modified Life-Space Assessment in Multimorbid Older Persons With Cognitive Impairment. *Gerontologist* 2019;**59**:e66-e75.

24 Auger C, Demers L, Gelinas I, et al. Development of a French-Canadian version of the Life-Space Assessment (LSA-F): content validity, reliability and applicability for power mobility device users. *Disability and rehabilitation Assistive technology* 2009;**4**:31-41.

25 Miyashita T, Tadaka E, Arimoto A. Cross-sectional study of individual and environmental factors associated with life-space mobility among community-dwelling independent older people. *Environmental Health and Preventive Medicine* 2021;**26**:9.

26 Tseng YC, Gau BS, Lou MF. Validation of the Chinese version of the Life-Space Assessment in community-dwelling older adults. *Geriatr Nurs* 2020;**41**:381-6.

27 Fristedt S, Kammerlind AS, Bravell ME, et al. Concurrent validity of the Swedish version of the life-space assessment questionnaire. *BMC Geriatr* 2016;**16**:181.

For peer review only

28 Pedersen MM, Kjaer-Sorensen P, Midtgaard J, et al. A Danish version of the life-space assessment (LSA-DK) - translation, content validity and cultural adaptation using cognitive interviewing in older mobility limited adults. *BMC Geriatr* 2019;**19**:312.

29 Hauer K, Ullrich P, Heldmann P, et al. Psychometric Properties of the Proxy-Reported Life-Space Assessment in Institutionalized Settings (LSA-IS-Proxy) for Older Persons with and without Cognitive Impairment. *International journal of environmental research and public health* 2021;**18**:3872.

30 Ullrich P, Eckert T, Bongartz M, et al. Life-space mobility in older persons with cognitive impairment after discharge from geriatric rehabilitation. *Archives of gerontology and geriatrics* 2019;**81**:192-200.

31 Auais M, Alvarado B, Guerra R, et al. Fear of falling and its association with life-space mobility of older adults: a cross-sectional analysis using data from five international sites. *Age Ageing* 2017;**46**:459-65.

32 Faul F, Erdfelder E, Buchner A, et al. Statistical power analyses using G*Power 3.1: tests for correlation and regression analyses. *Behavior research methods* 2009;**41**:1149-60.

33 WHO. Process of translation and adaption of instruments. https://www.who.int/substance_abuse/research_tools/translation/en/ 2008.

34 Forsyth BHK, Martha Stapelton; Lawrence, Deidre; Levine, Kerry; Willis Gorgon B. Methods for Translating Survey Questionaires. *American Association for Public Opinion Research* 2006.

35 Einig K, Zaspel-Heisters B. Das System Zentraler Orte in Deutschland. In: Flex F, Greiving S, eds. *Neuaufstellung des Zentrale-Orte Konzepts in Nordrein-Westfalen*. Hannover: Verlag des ARL 2016.

For peer review only

36 Ritchie CS, Locher JL, Roth DL, et al. Unintentional weight loss predicts decline in activities of daily living function and life-space mobility over 4 years among community-dwelling older adults. *J Gerontol A Biol Sci Med Sci* 2008;**63**:67-75.

37 Sheppard KD, Sawyer P, Ritchie CS, et al. Life-space mobility predicts nursing home admission over 6 years. *Journal of aging and health* 2013;**25**:907-20.

38 Borsch-Supan A, Brandt M, Hunkler C, et al. Data Resource Profile: the Survey of Health, Ageing and Retirement in Europe (SHARE). *Int J Epidemiol* 2013;**42**:992-1001.

39 Turner G, Clegg A. Best practice guidelines for the management of frailty: a British Geriatrics Society, Age UK and Royal College of General Practitioners report. *Age and Ageing* 2014;**43**:744-7.

40 Podsiadlo D, Richardson S. The timed "Up & Go": a test of basic functional mobility for frail elderly persons. *Journal of the American Geriatrics Society* 1991;**39**:142-8.

41 Lin MR, Hwang HF, Hu MH, et al. Psychometric comparisons of the timed up and go, one-leg stand, functional reach, and Tinetti balance measures in community-dwelling older people. *Journal of the American Geriatrics Society* 2004;**52**:1343-8.

42 Donoghue OA, Savva GM, Cronin H, et al. Using timed up and go and usual gait speed to predict incident disability in daily activities among community-dwelling adults aged 65 and older. *Archives of physical medicine and rehabilitation* 2014;**95**:1954-61.

43 Ludwig K, Graf von der Schulenburg JM, Greiner W. German Value Set for the EQ-5D-5L. *PharmacoEconomics* 2018;**36**:663-74.

44 Schott N. Reliability and validity of the German short version of the Activities specific Balance Confidence (ABC-D6) scale in older adults. *Archives of gerontology and geriatrics* 2014;**59**:272-9.

For peer review only

45 Buchner DM, Hornbrook MC, Kutner NG, et al. Development of the common data base for the FICSIT trials. *Journal of the American Geriatrics Society* 1993;**41**:297-308.

46 Sousa-Santos AR, Amaral TF. Differences in handgrip strength protocols to identify sarcopenia and frailty - a systematic review. *BMC Geriatr* 2017;**17**:238.

47 Charlson ME, Pompei P, Ales KL, et al. A new method of classifying prognostic comorbidity in longitudinal studies: development and validation. *Journal of chronic diseases* 1987;**40**:373-83.

48 Borson S, Scanlan J, Brush M, et al. The mini-cog: a cognitive 'vital signs' measure for dementia screening in multi-lingual elderly. *International journal of geriatric psychiatry* 2000;**15**:1021-7.

49 Hesterberg TMDSM, S. ; Clipson, A. ; Epstein, R. Bootstrap methods and permutation tests. In: A. MDSMGPCB, ed. *Intoduction to practice of statistics*. New York: Palgrave Macmillan 2007.

50 Rasch D, Kubinger KD, Moder K. The two-sample t test: pre-testing its assumptions does not pay off. *Statistical Papers* 2011;**52**:219-31.

1

For peer review only

Footnotes

Contributors: JK developed the first draft of the study. JK, SAM and PG planned and conducted the final studyprotocol. JK and SAM translated LSA into German, compiled the final version of the LSA-D, and contributed in recruiting and testing participants. SAM drafted the manuscript and conducted the major analyses. MS drafted tables and the figure. PG established the statistical analysis plan and performed the sample size and power calculation. MS assisted with data analyses and interpretation of the results. JLOS gave advice and commented on the manuscript. All authors critically reviewed the manuscript and approved the final version.

Funding: The study was partly financed by the "MOBILE"-Project funded by the Federal Ministry of Education and Research (BMBF; ID 01GY1803) and the Institute of Medical Sociology and Rehabilitation Science, Charité – Universitätsmedizin Berlin. We acknowledge support from the German Research Foundation (DFG) and the Open Access Publication Fund of Charité – Universitätsmedizin Berlin.

Competing Interests: Non declared.

Patient consent for publication: Not required

Patient and public involvement: Patients and/or the public were not involved in the design, or conduct, or reporting, or dissemination plans of this research.

Ethical approval: Ethics approval for the study was provided by the Ethics Committee of Charité – Universitätsmedizin Berlin (Rec. Reference EA2/124/19).

Data availability statement: Data will be made available on reasonable, methodologically sound request as soon as possible to achieve the aims of the approved proposal.

Available data includes individual participant data that underlies the results reported in this article after its deidentification, data dictionary and the analytical code.

Data will be available at least 3 months after, and ending 5 years following article publication.

Proposals should be directed at sandra.muemken@charite.de or joern.kiselev@charite.de

For peer review only

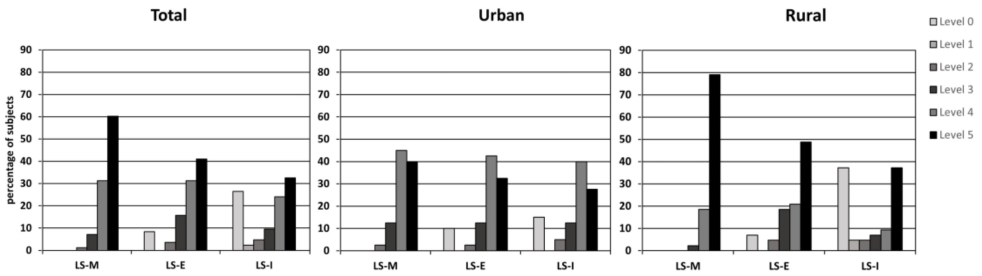

Distribution of different life-space levels of the LSA-D among the total sample, urban and rural subsample.
LS-M = maximal life-space; LS-E = life-space with equipment: LS-I = independent life-space

209x69mm (400 x 400 DPI)

German Life-Space-Assessment LSA-D

DIE NÄCHSTEN FRAGEN BEZIEHEN SICH AUF IHRE AKTIVITÄTEN IN DEN LETZTEN VIER WOCHEN:			A. WIE HÄUFIG WAREN SIE IN DEN LETZTEN 4 WOCHEN IN (Name des Life-Space-Levels)? Häufigkeit				WIE SIND SIE DORT HINGEKOMMEN? B. HABEN SIE HILFSMITTEL ODER ANDERE AUSRÜSTUNG DAFÜR VERWENDET?			WIE SIND SIE DORT HINGEKOMMEN? C. BENÖTIGTEN SIE DAFÜR DIE HILFE EINER ANDEREN PERSON?		
WAREN SIE IN DEN LETZTEN 4 WOCHEN…	Ja	Nein	Weniger als 1 mal die Woche	1-3 mal die Woche	4-6 mal die Woche	Täglich	Ja	Nein	Unbekannt / keine Angabe	Ja	Nein	Unbekannt / keine Angabe
IN ANDEREN RÄUMEN IHRES ZUHAUSES außer dem Raum, in dem Sie schlafen? *LIFE-SPACE 1*	**LS1** ❑	❑	**LS1-Häufigkeit** ❑	❑	❑	❑	**LS1-Hilfsmittel** ❑	❑	❑	**LS1-Persönliche Hilfe** ❑	❑	❑
IN DER NÄHEREN UMGEBUNG AUSSERHALB IHRER WOHNUNG (Hausflur, Terrasse, Balkon, Fahrstuhl, Hof, Garage, hauseigener Garten, Auffahrt)? *LIFE-SPACE 2*	**LS2** ❑	❑	**LS2-Häufigkeit** ❑	❑	❑	❑	**LS2-Hilfsmittel** ❑	❑	❑	**LS2-Persönliche Hilfe** ❑	❑	❑
AN ORTEN IN IHRER NACHBARSCHAFT, aber außerhalb Ihrer Wohnung oder Ihrer näheren Wohnumgebung? *LIFE-SPACE 3*	**LS3** ❑	❑	**LS3-Häufigkeit** ❑	❑	❑	❑	**LS3-Hilfsmittel** ❑	❑	❑	**LS3-Persönliche Hilfe** ❑	❑	❑
AN ORTEN AUSSERHALB IHRER NACHBARSCHAFT, aber innerhalb der Stadt oder der Ortschaft, in der Sie leben? *LIFE-SPACE 4*	**LS4** ❑	❑	**LS4-Häufigkeit** ❑	❑	❑	❑	**LS4-Hilfsmittel** ❑	❑	❑	**LS4-Persönliche Hilfe** ❑	❑	❑
AN ORTEN AUSSERHALB DER STADT ODER DER ORTSCHAFT, in der Sie leben? *LIFE-SPACE 5*	**LS5** ❑	❑	**LS5-Häufigkeit** ❑	❑	❑	❑	**LS5-Hilfsmittel** ❑	❑	❑	**LS5-Persönliche Hilfe** ❑	❑	❑

For peer review only
